# Assessment of cognitive performance in multiple sclerosis using smartphone-based training games: a feasibility study

**DOI:** 10.1007/s00415-023-11671-9

**Published:** 2023-03-23

**Authors:** Silvan Pless, Tim Woelfle, Yvonne Naegelin, Johannes Lorscheider, Andrea Wiencierz, Óscar Reyes, Pasquale Calabrese, Ludwig Kappos

**Affiliations:** 1 Research Center for Clinical Neuroimmunology and Neuroscience Basel (RC2NB), University Hospital Basel, University of Basel, Spitalstrasse 2, 4031 Basel, Switzerland; 2 Department of Neurology, University Hospital Basel, Basel, Switzerland; 3 Neuropsychology and Behavioral Neurology Unit, Department of Psychology and Interdisciplinary Platform Psychiatry and Psychology, Division of Molecular and Cognitive Neuroscience, University of Basel, Basel, Switzerland; 4 Clinical Trial Unit, University Hospital Basel, Basel, Switzerland; 5 Healios AG, Basel, Switzerland

**Keywords:** Multiple sclerosis, Cognitive assessment, Gamification, Monitoring-tool, Smartphone-games

## Abstract

**Background:**

Cognitive impairment occurs in up to 70% of people with MS (pwMS) and has a large impact on quality of life and working capacity. As part of the development of a smartphone-app (dreaMS) for monitoring MS disease activity and progression, we assessed the feasibility and acceptance of using cognitive games as assessment tools for cognitive domains.

**Methods:**

We integrated ten cognitive games in the dreaMS app. Participants were asked to play these games twice a week for 5 weeks. All subjects underwent a battery of established neuropsychological tests. User feedback on acceptance was obtained via a five-point Likert-scale questionnaire. We correlated game performance measures with predetermined reference tests (Spearman’s rho) and analyzed differences between pwMS and Healthy Controls (rank biserial correlation).

**Results:**

We included 31 pwMS (mean age 43.4 ± 12.0 years; 68% females; median Expanded Disability Status Scale score 3.0, range 1.0–6.0) and 31 age- and sex-matched HC. All but one game showed moderate–strong correlations with their reference tests, (|*r*_*s*_*|*= 0.34–0.77). Performance improved in both groups over the 5 weeks. Average ratings for overall impression and meaningfulness were 4.6 (range 4.2–4.9) and 4.7 (range 4.5–4.8), respectively.

**Conclusion:**

Moderate–strong correlations with reference tests suggest that adaptive cognitive games may be used as measures of cognitive domains. The practice effects observed suggest that game-derived measures may capture change over time. All games were perceived as enjoyable and meaningful, features crucial for long-term adherence. Our results encourage further validation of adaptive cognitive games as monitoring tools for cognition in larger studies of longer duration.

**Study Register:**

ClinicalTrials.gov: NCT04413032.

**Supplementary Information:**

The online version contains supplementary material available at 10.1007/s00415-023-11671-9.

## Introduction

Multiple Sclerosis (MS) a chronic inflammatory and degenerative autoimmune disease of the central nervous system affecting approximately 2.4 million people worldwide is one of the most prevalent neurological causes of permanent disability in young adults [1, 2]. Up to 70% of people with Multiple Sclerosis (pwMS) show signs of cognitive impairment (CI) [3]. These symptoms affect emotional well-being, working capacity and quality of life (QoL) [4, 5]. Still, because comprehensive neuropsychological test batteries are time-consuming, require specialized examiners, and are not always well accepted by patients’ systematic cognitive assessments are not widely used in clinical practice, even in specialized centers [6]. To better understand disease progression and optimize treatment strategies, a more complete and detailed monitoring of cognitive functions is desirable.

In recent years, there have been many advances in digitization of cognitive assessment using the potential of digital devices such as computers, tablets, and smartphones [7–14]. Especially smartphone apps have many advantages: they are easily available, user-friendly and cheap, making them the ideal tool to reach a large range of the population [15]. Importantly, recent research has shown that digital games can offer valuable information about cognitive functions [7]. Thus, we hypothesized that adaptive smartphone-games may have relevant advantages over the standard neuropsychological tests regarding acceptance and motivation, accuracy (e.g. more accurate measurement through touchscreen-sensors vs. test-rater using stopwatch), standardization and objectivity (no rater-bias) and convenience (accessibility of smartphones) [12, 13, 16].

In this proof-of-concept study, we correlate measures derived from adaptive smartphone-based cognitive training games with results of predefined corresponding established cognitive paper and pencil tests. The primary objective is to identify cognitive game measures that correlate with established neuropsychological tests and identify games that are well-accepted and meaningful for pwMS. Additional exploratory objectives were (a) investigating correlations of all reference test scores with all game measure scores; (b) determining whether the games and tests could be attributed to specific cognitive domains, and c) analyzing differences between pwMS and HC regarding game performance and game ratings.

### Methods

The Research Center for Clinical Neuroimmunology and Neuroscience Basel (RC2NB) is currently developing a smartphone app „dreaMS “, in cooperation with the medical device software manufacturer Healios AG [17, 18]. This app aims to allow monitoring of a large variety of potential digital biomarkers by having patients repeatedly perform short tasks via their smartphone. To reach the largest population possible, the app works on a wide range of smartphone models with both android (5.0 (API 21) or later) and iOS (11.0 or later) operating systems. The tasks included in the dreaMS app cover five different functional domains: dexterity, walking ability, balance, cognition, and vision. Additionally, the app includes patient-reported outcome measures (PROs) for fatigue, walking ability, general symptoms, and quality of life [19]. As part of a study to investigate the technical feasibility, reliability, and acceptance of the dreaMS app (NCT04413032), we integrated 10 adaptive cognitive training games from the commercially available brain training app Peak [20] in the dreaMS app via deep link.

Here, we report on the performance of these cognitive games as measures of cognitive domains including their correlation with established neuropsychological tests, acceptance by users, and assessment of their meaningfulness by pwMS.

### Standard protocol approvals, registrations, and patient consents

This study was approved by the local ethics committee (Ethikkommission Nordwest- und Zentralschweiz (EKNZ), Basel, Switzerland, on July 17th 2020/project-ID 2020-01515). All participants gave their written informed consent. This study conforms with World Medical Association Declaration of Helsinki and was registered at ClinicalTrials.gov: NCT04413032.

### Study procedures

Participants attended three visits, all of them taking place at the MS Center at the University Hospital Basel: 1. Screening visit (S), 2. Baseline visit (BL) and 3. End-of-study visit (EoS). A written informed consent was obtained from all participants at the screening visit. At BL, all participants underwent a neurological examination and completed a battery of established standardized neuropsychological tests (Table 1). The participants were instructed to download the dreaMS app which included the Peak games. A study nurse went through every task with the participants to ensure their understanding. During the 6-week study period, the participants were asked to play each game twice a week at home during week 1–5, according to a given schedule. To ensure maximum adherence, the participants received automated messages multiple times a week, reminding them to complete the tasks. After completion of a game, the data were uploaded and stored on a secure server of the University Hospital Basel. Every completion of a game was registered in the study portal, where study nurses were able to check adherence and, if necessary, contact the participant personally to remind them to complete the tasks. The participants then had the opportunity to complete the games behind schedule, provided they did so in the same week. In week 6, participants were asked to fill out multiple PROs via the app. At EoS, we collected participants’ feedback regarding acceptance and meaningfulness via a five-point Likert scale questionnaire. The complete feedback questionnaire can be found in the supplementary material (S1: Feedback questionnaire).

**Table 1:** Cognitive games, targeted cognitive domains, and their corresponding reference tests

Cognitive game	Cognitive domain	Reference test
Word hunt	Language	Regensburger Wortflüssigkeitstest (RWT)^a^ [24]
Spin cycle	Working memory	Symbol Digit Modalities Test (SDMT) [25]
Zap gap	Inhibition	Stroop test [26] Incongruence sub-test
Face switch	Inhibition	Stroop test Incongruence sub-test
Rush back	Working memory	Symbol Digit Modalities Test (SDMT)
Baggage claim	Short-term memory	Verbaler Lern- und Merkfähigkeitstest (VLMT)^b^ [27] Learning-trials 1–5 subtest
Perilous path	Visuo-spatial short-term memory	Rey-Osterrieth Complex Figure Test (ROCF) [28] 3-min recall sub-test
Puzzle blox	Visuo-spatial construction	Rey-Osterrieth Complex Figure Test (ROCF) Copy sub-test
Must sort	Processing speed	Symbol Digit Modalities Test (SDMT)
Low pop	Mental flexibility	Trail Making Test A&B (TMT A&B) [29]

^a^RWT = German word-fluency test

^b^VLMT = German verbal learning- and memory test

### Participants

Inclusion criteria for pwMS comprised a diagnosis of MS (RRMS, SPMS, PPMS, CIS) according to the revised McDonald criteria (2017) [21] and an Expanded Disability Status Scale (EDSS) of ≤ 6.5. Further, clinical stability at the time of inclusion and during the whole study period was a prerequisite (pwMS who experienced a relapse or clinical progression during the study period would be excluded from the study). Both pwMS and HC were required to be between 18 and 70 years old, to own and be capable of using a smartphone device fulfilling defined minimum technical standards, have sufficient dexterity and visual functions, and be able to follow the study procedures. The complete list of in- and exclusion criteria can be found in the supplementary material (S2: Inclusion and exclusion criteria).

### Instruments and measurements

10 cognitive training games from Peak were included in the dreaMS app via deep link [20]. Game selection was based on domains known to be relevant in pwMS [4]: short-term and working memory, mental flexibility and processing speed, inhibition, language, and visuo-construction. All games were structured with multiple difficulty levels, which would adapt to the performance. This difficulty-adaptation is based on the sequence of correct/false answers: after X consecutive correct answers, the difficulty increases, after X consecutive errors, the difficulty decreases. Furthermore, a ranking-system is included: if the user reaches a certain score-threshold in two consecutive gaming sessions, the following session will start from a higher difficulty. Equally, the same is the case for leveling down. For each game, we determined quantifiable features as measures of game performance. Features are measures derived from the results of a test. Typically, in cognitive tests, these are: *Number of correct answers within a given time frame* but also *number of errors* per se. Depending on the structure of a cognitive game additional features like qualitative and temporal patterns of errors might also be derived or a combination of such features. In adaptive games, the *level of difficulty reached* by the participant could also by itself be a valid measure. We prospectively assigned each game to a cognitive domain according to information provided by the game developer and expert consensus. Every Peak game was intended to train a specific cognitive domain, which was developed under supervision of certified neuropsychologists at Peak [20]. To ensure the correctness of this categorization, P. Calabrese and S. Pless analyzed the tasks of each game and confirmed that they represent the domain proposed by the developer. Established cognitive tests covering the same cognitive domains were then selected out of a comprehensive neuropsychological test battery used routinely in the assessment of pwMS participating in the Swiss MS Cohort Study (SMSC) [22]. Regarding the selection of game-features, we focused on quantifiable measures most similar to those used in established cognitive assessments e.g. *the number of correct answers in a given time interval*, since it includes both speed and accuracy. However, the games have the additional feature of difficulty-adaptation, according to a scoring system based on the number of correct answers. Since the difficulty level adapts, the measure *number of correct answers* can be misleading for some games. In these cases, taking the change in difficulty level itself as the measure was the preferred option (table S1: Description of cognitive games and measures used for statistical analyses).

As a general screening test of cognitive impairment we used the MUSIC (Multiple Sclerosis Inventory of Cognition) [23]. The cognitive games chosen, their corresponding reference tests, and the cognitive domain they refer to, are shown in Table 1. The game-derived measures used for the statistical analyses and brief game descriptions are listed in the supplementary material (table S1: Description of cognitive games and measures used for statistical analyses). Exemplary Screenshots of the Peak Games are shown in the supplementary material (figures S1–S6: Exemplary Screenshots of Peak Games).

At end of study (EoS), all participants were asked to provide feedback. For each game, participants rated four questions on a five-point Likert scale. The questions addressed the participant’s overall impression, whether the game was appropriately challenging (including an additional elaboration question), whether the participant would be willing to play the games regularly in the future, and whether they thought the games were relevant for MS (meaningfulness). The question about meaningfulness was only asked to pwMS. Mean ratings of those four questions were used to calculate overall acceptance.

### Objectives, outcomes and statistical analyses

The primary objective was to identify cognitive game measures that correlated with established neuropsychological tests and identify games that are well-accepted and meaningful for pwMS. Hence, the primary study outcomes were (a) correlation coefficient between game measures and the corresponding reference test scores. The average of the game measures over all ten sessions (2x/week for 5 weeks) was correlated with the corresponding established neuropsychological reference test score using spearman’s rank correlation coefficient. As an acceptable threshold, we targeted an at least moderate (*r*_*s*_ ≥ 0.3) correlation coefficient [30]; (b) user acceptance and meaningfulness of the games for pwMS as assessed via a 5-point Likert scale questionnaire. Regarding user acceptance (overall impression, meaningfulness, and willingness to use in the future) we aimed for a mean Likert scale score of ≥ 3.

Additional exploratory objectives were the correlations of all reference test scores (mean) with all game measure scores (mean), using spearman’s rank correlation coefficient in correlation matrices. Further, we investigated whether the games and tests could be attributed to specific cognitive domains by performing a factor analysis including all game measures (mean) and test scores (mean), using maximum likelihood estimation and the rotation method “varimax”. Differences between pwMS and HC regarding game performance were assessed by means of rank biserial correlations between the average game scores over all ten sessions and the group variable. Since the analysis of differences between the two groups was exploratory, we used the more conservative analysis method: rank biserial correlations, in order to avoid misinterpretation. Spaghetti plots of all participants’ game performances over the ten sessions were created to better visualize group differences over time. Further, we compared the mean Likert scale game ratings across pwMS and HC using rank biserial correlation.

Statistical analyses were performed using the Statistical Package for the Social Sciences (SPSS).

Version 28.0 and R version 4.2.0 (2022-04-22, R Core Team, 2022). The study protocol and statistical analysis plan are available as supplementary material (S3: Study protocol).

### Data access and availability statement

Ludwig Kappos, Silvan Pless, and Andrea Wiencierz take full responsibility for the data, the analyses and interpretation, and the conduct of the research, have full access to all of the data and have the right to publish any data separate and apart from any sponsor. The data supporting the findings of this study are available from the corresponding author upon reasonable request.

## Results

Between October 5th 2020 and February 28th 2021, we recruited 31 pwMS from the MS Center, University Hospital Basel with a mean age of 43.4 ± 12.0 years, 68% females with a median Expanded Disability Status Scale score of 3.0 (range 1.0–6.0) and 31 age- and sex-matched HC. Table 2 provides an overview of the participants’ demographics. Three participants (2 HC, 1 pwMS) were excluded from the performance analyses due to incomplete data, however, they were included in the acceptance rating analysis.

**Table 2:** Demographics of study participants

	People with MS (*n = *31)	Healthy controls (*n = *31)
Mean age in years (SD)	43.4 (± 12)	42.8 (± 11.9)
Gender female, n (%)	21 (68%)	21 (68%)
MS type, *n* (%)		–
CIS	2 (6%)	
RRMS	23 (74%)	
SPMS	2 (6%)	
PPMS	4 (13%)	
Median EDSS (range)	3.0 (1.0–6.0)	–
Treatment, *n* (%)		–
Untreated	6 (19%)	
Interferon beta	1 (3%)	
Glatiramer acetate	1 (3%)	
Teriflunomide	3 (10%)	
Dimethyl fumarate	1 (3%)	
Fingolimod	7 (22%)	
Natalizumab	2 (6%)	
Rituximab	1 (3%)	
Ocrelizumab	9 (29%)	
MUSIC results	(*n = *30)	(*n = *29)
Mean score (SD)	24 (± 4.9)	26 (± 3.9)
Median score	23	27
Distribution by CI group, *n* (%)		
No CI (score 20–32)	23 (74.2%)	29 (100%)
Mild CI (score 16–19)	4 (12.9%)	0 (0%)
Moderate CI (score 11–15)	3 (9.7%)	0 (0%)
Severe CI (score – 3 to 10)	0 (0%)	0 (0%)

According to the results of MUSIC cognitive impairment (CI) was present in 7/30 (22.5%) pwMS (3 (9.7%) moderate and 4 (12.9%) mild). No CI was observed in the HC group. When comparing the mean MUSIC score between pwMS (24 ± 4.9) and HC (26 ± 3.9), no clear differences were found (|*r*_rb_|= − 0.24, *p* = 0.07). We did not find a strong correlation between the MUSIC and the EDSS (*r*_*s*_ = − 0.33, 95%CI − 0.62 to 0.05, *p* = 0.08).

CIS clinically isolated syndrome; *RRMS* relapse remitting MS; *SPMS* secondary progressive MS; *PPMS* primary progressive MS; *EDSS* expanded disability status scale; *MUSIC* multiple sclerosis inventory of cognition; *CI* cognitive impairment

### Primary outcomes

#### Correlation of game-derived measures with established neuropsychological tests

All but the language-game *Word Hunt* (*r*_*s*_ = − 0.25, 95%CI − 0.48 to 0.01) reached the preset level of moderate spearman correlation coefficients (|*r*_*s*_*|*≥ 0.3) with their predefined reference tests. Six games (*Must Sort* (*r*_*s*_ = 0.77, 95%CI 0.64–0.86), *Spin Cycle* (*r*_*s*_ = 0.53, 95%CI 0.31–0.70), *Rush Back* (*r*_*s*_ = 0.66, 95%CI 0.48–0.79), *Face Switch* (*r*_*s*_ =− 0.51, 95%CI − 0.68 to − 0.28), *Perilous Path* (*r*_*s*_ = 0.51, 0.28–0.68), and *Low Pop* (*r*_*s*_ = − 0.72, 95%CI − 0.82 to − 0.56) correlated strongly with their reference tests. Three (*Zap Gap* (*r*_*s*_ =− 0.34, 95%CI − 0.55 to − 0.08), *Baggage Claim* (*r*_*s*_ = 0.48, 95%CI 0.25–0.67), and *Puzzle Blox* (*r*_*s*_ = 0.44, 95%CI 0.20–0.63)) showed moderate correlation coefficients. An overview of the correlation coefficients, their 95% confidence intervals and p-values is shown in Table 3.

**Table 3:** Correlations between game measures and selected reference tests scores

Cognitive Domain	Game	Measure	Reference Test	Spearman's rho (95% CI), *p* value
Processing speed	Must sort	Number of successful rounds	SDMT	0.77 (0.64–0.86), < 0.01
Working memory	Spin cycle	Difficulty-level reached	SDMT	0.53 (0.31–0.70), < 0.01
	Rush back	Number of successful rounds	SDMT	0.66 (0.48–0.79), < 0.01
Inhibition	Zap gap	Difficulty-level reached	Stroop	− 0.34 (− 0.55 to − 0.08), < 0.01
	Face switch	Number of successful rounds	Stroop	− 0.51 (− 0.68 to − 0.28), < 0.01
Short-term memory	Baggage claim	Difficulty-level reached	VLMT	0.48 (0.25–0.67), < 0.01
	Perilous path	Difficulty-level reached	ROCF-recall	0.51 (0.28–0.68), < 0.01
Visuo-construction	Puzzle blox	Difficulty-level reached	ROCF-copy	0.44 (0.20–0.63), < 0.01
Mental flexibility	Low pop	Difficulty-level reached	TMT B	− 0.72 (− 0.82 to − 0.56), < 0.01
Language	Word hunt	Completion time (s)	RWT	− 0.25 (-0.48 to 0.01), 0.06

*SDMT* symbol digit modalities test; *VLMT*  Verbaler Lern-und Merkfähigkeitstest (german verbal learning and memory test); *ROCF* rey-osterrieth complex figure test; *TMT B* Trail Making Test B; *RWT* Regensburger Wortflüssigkeitstest (german verbal fluency test)

#### Acceptance and meaningfulness

Over all games, the mean Likert scale rating was 4.63 (range 4.15–4.9) for *overall impression*, 4.40 (range 4.13–4.58) for *perceived difficulty*, 4.50 (range 4.15–4.77) for *willingness to use in the future* and 4.69 (range: 4.52–4.84) for *meaningfulness for pwMS*. All categories in all games met the predefined target of ≥ 3. Table 4 shows the means and standard deviations of acceptance ratings by game.

**Table 4:** Mean (SD) ratings by game (acceptance: all participants; meaningfulness: pwMS)

Domain	Game	Overall impression (*n = *62)	Perceived difficulty (*n = *62)	Future use (*n = *62)	MS-relevance (*n = *31)
Processing speed	Must Sort	4.71^b^ (± 0.64)	4.32^a^ (± 1.10)	4.47^a^ (± 0.95)	4.73^b^ (± 0.52)
Working memory	Spin Cycle	4.15^a^ (± 0.99)	4.19^a^ (± 0.87)	4.15^a^ (± 1.14)	4.52^b^ (± 0.72)
	Rush Back	4.66^b^ (± 0.65)	4.32^a^ (± 1.02)	4.42^a^ (± 1.02)	4.68^b^ (± 0.70)
Inhibition	Zap Gap	4.18^a^ (± 1.08)	4.13^a^ (± 1.00)	4.16^a^ (± 1.03)	4.53^b^ (± 0.73)
	Face Switch	4.68^b^ (± 0.67)	4.40^a^ (± 0.98)	4.51^b^ (± 0.92)	4.68^b^ (± 0.65)
Short-term memory	Baggage Claim	4.71^b^ (± 0.73)	4.48^a^ (± 0.92)	4.60^b^ (± 0.80)	4.74^b^ (± 0.58)
	Perilous Path	4.84^b^ (± 0.58)	4.56^b^ (± 0.76)	4.73^b^ (± 0.71)	4.84^b^ (± 0.37)
Visuo-construction	Puzzle Blox	4.65^b^ (± 0.79)	4.48^a^ (± 0.84)	4.58^b^ (± 0.76)	4.68^b^ (± 0.60)
Mental flexibility	Low Pop	4.84^b^ (± 0.45)	4.56^b^ (± 0.86)	4.65^b^ (± 0.70)	4.74^b^ (± 0.51)
Language	Word Hunt	4.90^b^ (± 0.35)	4.58^b^ (± 0.88)	4.77^b^ (± 0.64)	4.81^b^ (± 0.40)
Mean over all games		4.63^b^	4.40^a^	4.50^b^	4.69^b^

^a^Mean rating ≥ 4.0

^b^Mean rating ≥ 4.5

### Exploratory outcomes

#### Correlation matrix and factor analysis

Of the 70 possible correlations (10 cognitive games * 7 reference tests), we found 2 near zero (|*r*_*s*_|< 0.1), 17 weak (|*r*_*s*_|≥ 0.1), 28 moderate (|*r*_*s*_|≥ 0.3), and 23 strong (|*r*_*s*_|≥ 0.5) correlations as shown in Table 5. Regarding correlations of the established reference tests among each other (21 possible combinations), 7 weak, 6 moderate, and 8 strong (Table 5). The exploratory factor analysis showed that separating the games and tests into four factors was sufficient. The *SDMT* showed equal factor loadings for two factors (factor 1 and 3). Attributing every game and test to the factor with its highest factor loading, factor 1 includes 2 tests (*SDMT* and *VLMT*), and 3 games (*Face Switch, Must Sort, and Rush Back*). One Test (*ROCF recall*) and 5 games (*Baggage Claim, Puzzle Blox, Perilous Path*, *Spin Cycle, and Zap Gap*) showed the highest factor loadings with factor 2. Four tests: *ROCF copy, Stroop, TMT b, SDMT,* and the game *Word Hunt* had the highest association with factor 3. Lastly, the test *RWT* and the game *Low Pop* showed the highest factor loadings with factor 4 (Table 6).

**Table 5:**
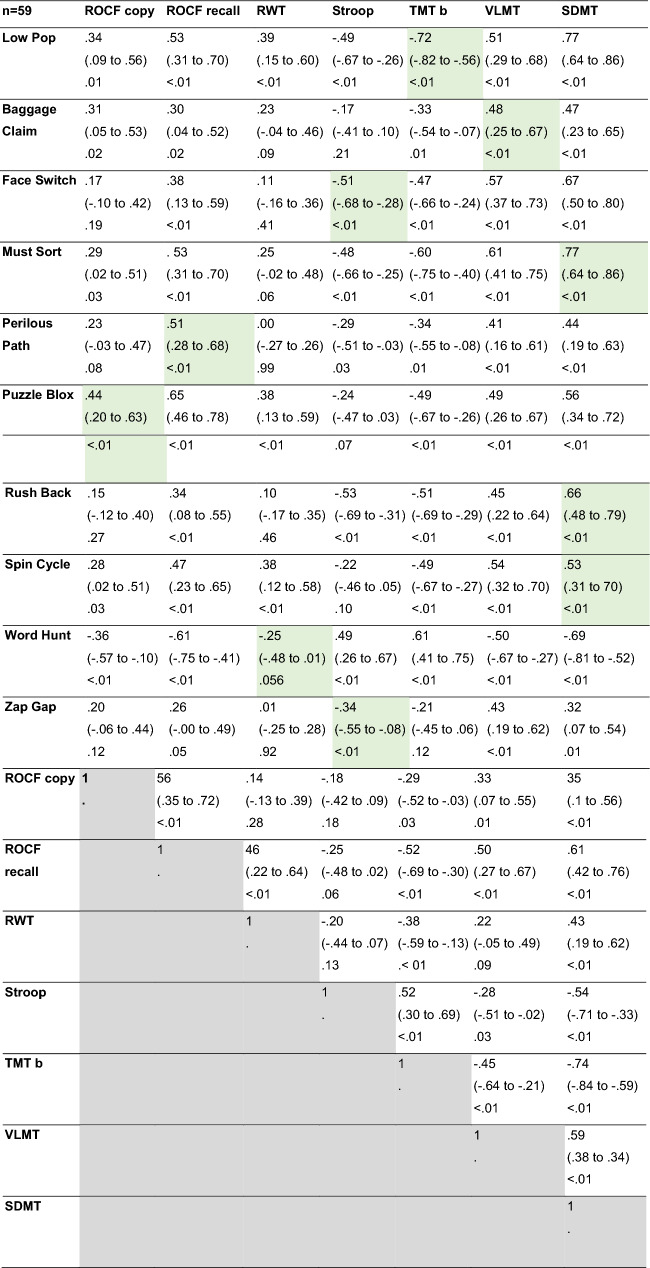
Correlation-matrix across game derived and reference test measures (Spearman’s rho, 95% confidence interval, and p-value) of all evaluable participants

Green shading indicates the values for the predefined game measure–reference test pairs

*SDMT* symbol digit modalities test; *VLMT*  Verbaler Lern-und Merkfähigkeitstest (german verbal learning and memory test); *ROCF* rey-osterrieth complex figure test; *TMT B* trail making test B; *RWT* Regensburger Wortflüssigkeitstest (german verbal fluency test)

**Table 6:**
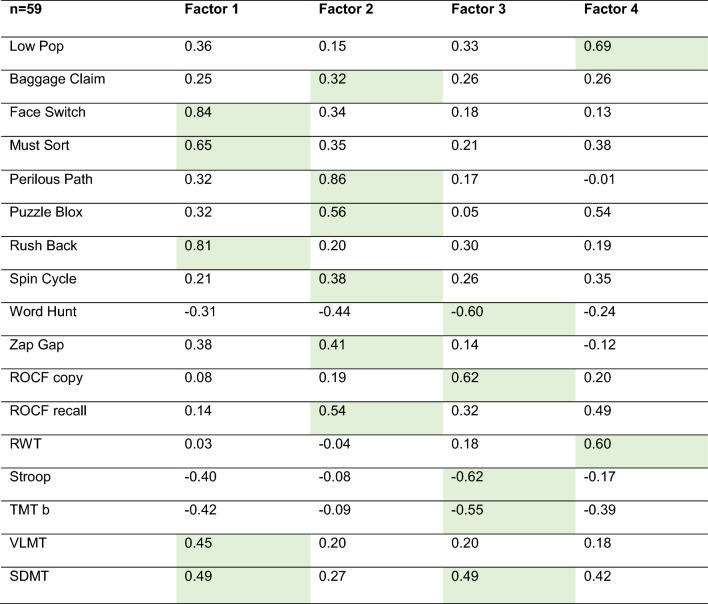
Factor analysis of games and reference tests (Maximum likelihood estimation, rotation method “varimax”) of all evaluable participants

Green shading indicates the highest factor loading

*SDMT* symbol digit modalities test; *VLMT*  Verbaler Lern-und Merkfähigkeitstest (german verbal learning and memory test); ROCF rey-osterrieth complex figure test; *TMT B* trail making test B; *RWT* Regensburger Wortflüssigkeitstest (german verbal fluency test)

#### Group differences in game performance

**Fig. 1:**
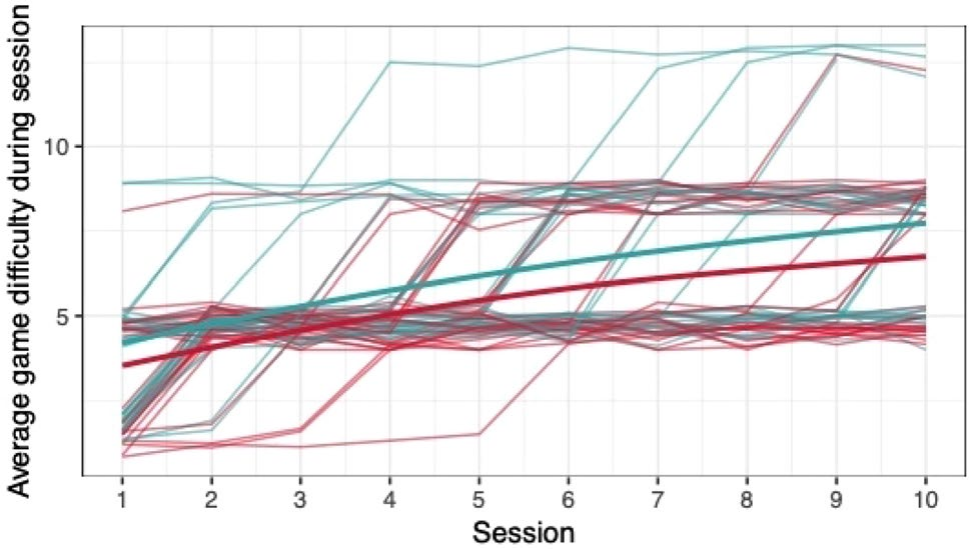
Individual performance in low pop

**Fig. 2:**
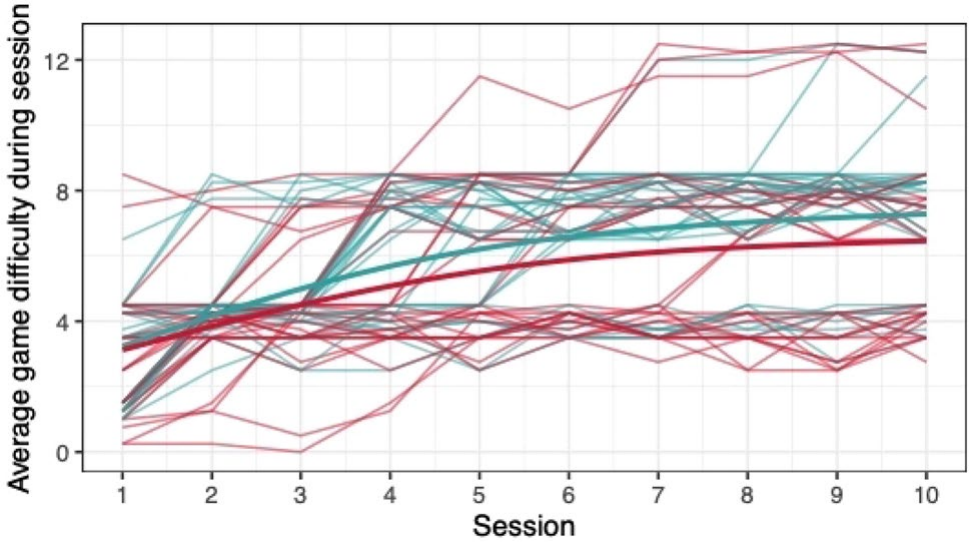
Individual performance in baggage claim

**Fig. 3:**
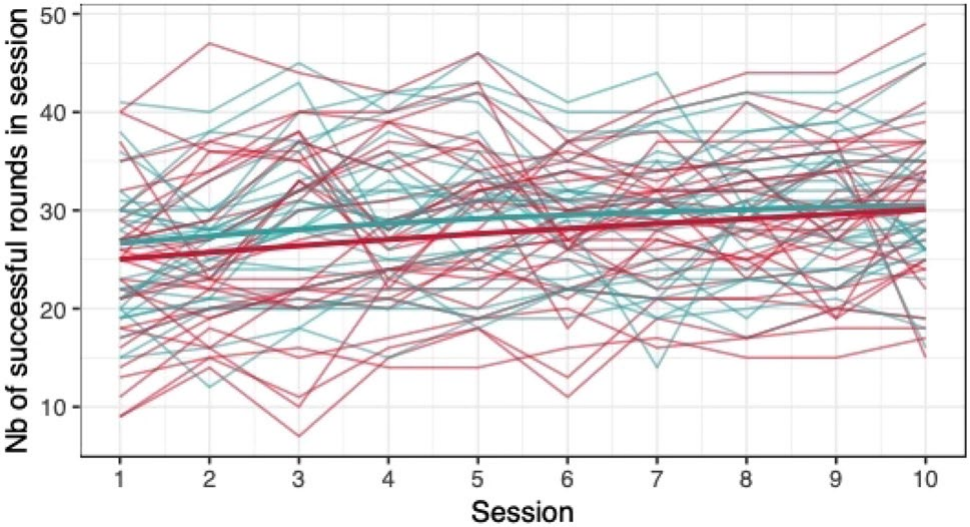
Individual performance in face switch

**Fig. 4:**
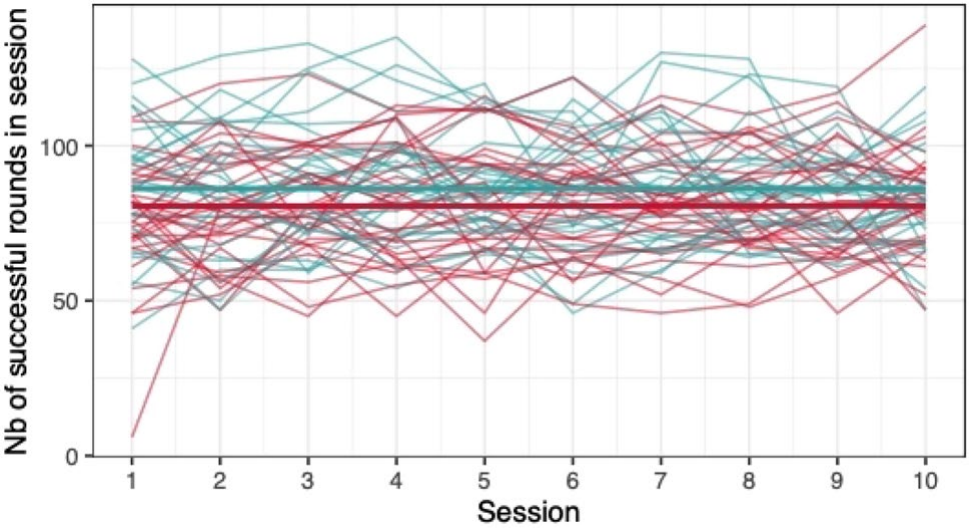
Individual performance in must sort

**Fig. 5:**
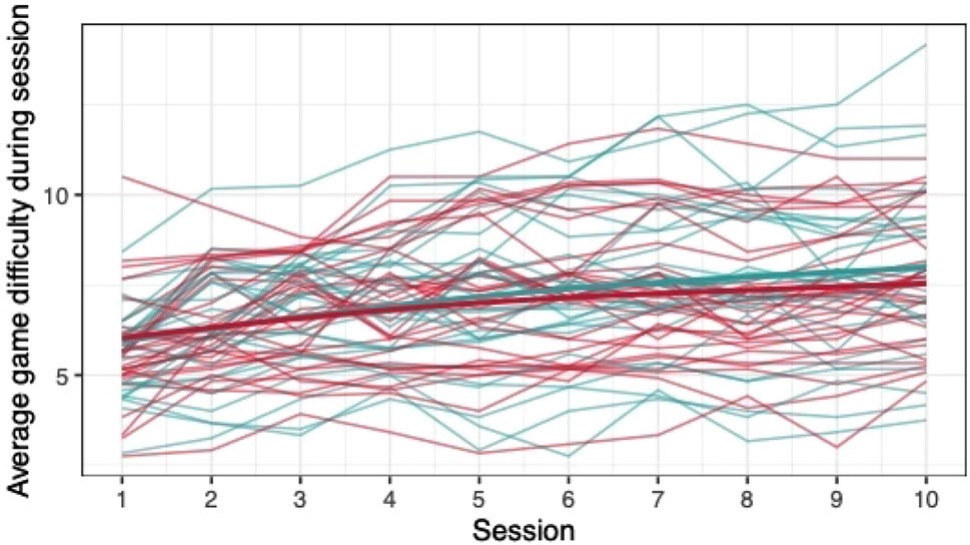
Individual performance in perilous path

**Fig. 6:**
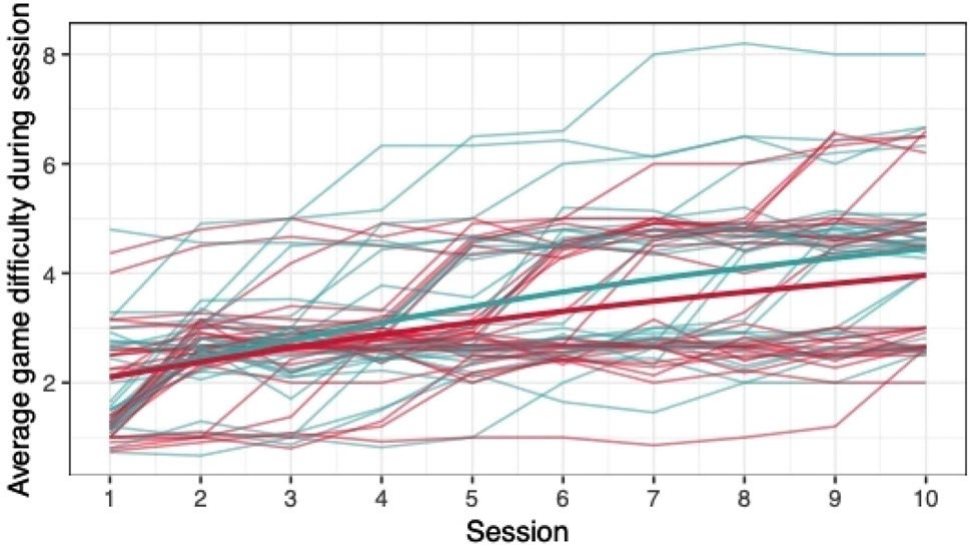
Individual performance in puzzle blox

**Fig. 7:**
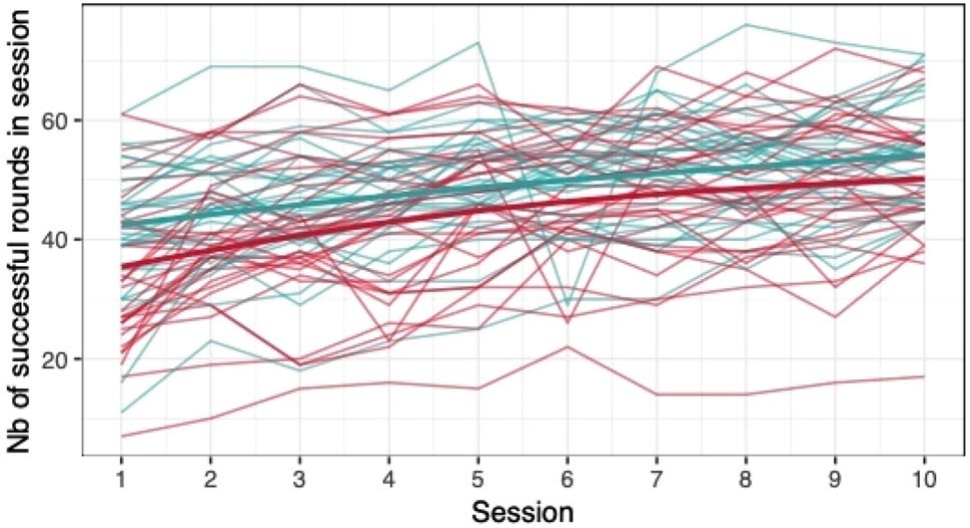
Individual performance in rush back

**Fig. 8:**
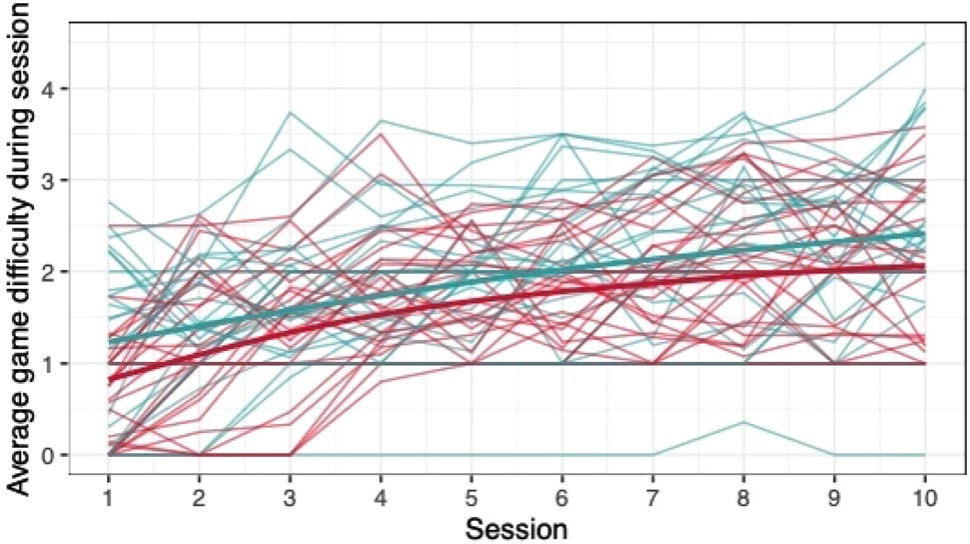
Individual performance in spin cycle

**Fig. 9:**
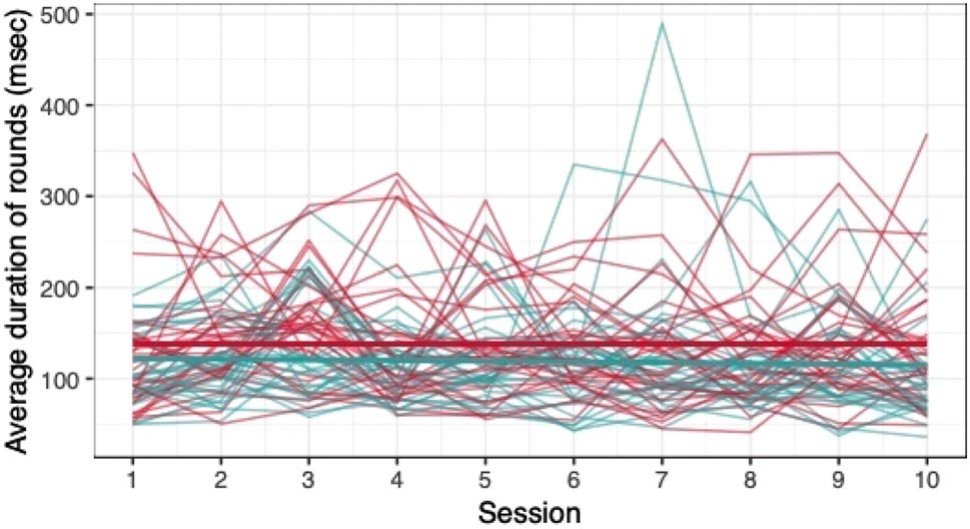
Individual performance in word hunt

**Fig. 10:**
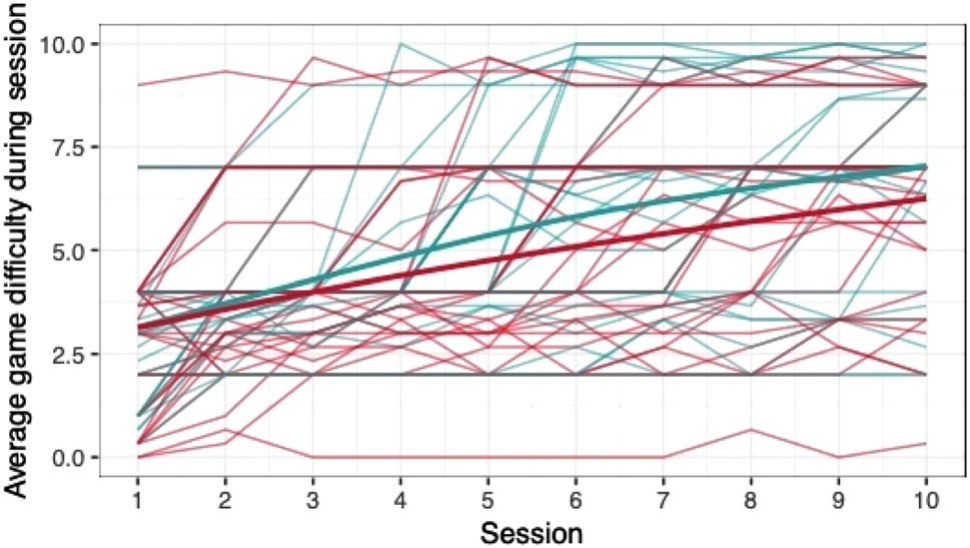
Individual performance in zap gap

**Fig. 11:**
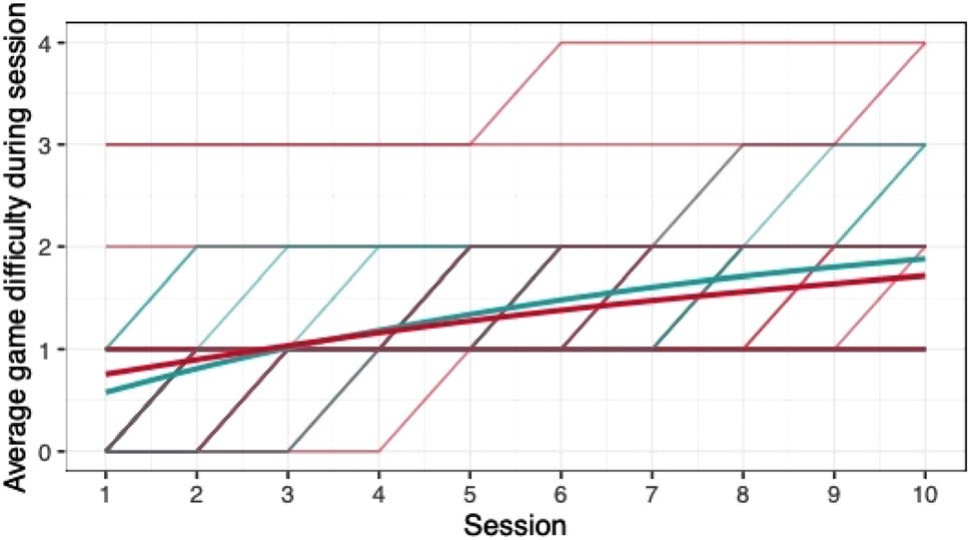
Alternative measure in must sort

**Fig. 12:**
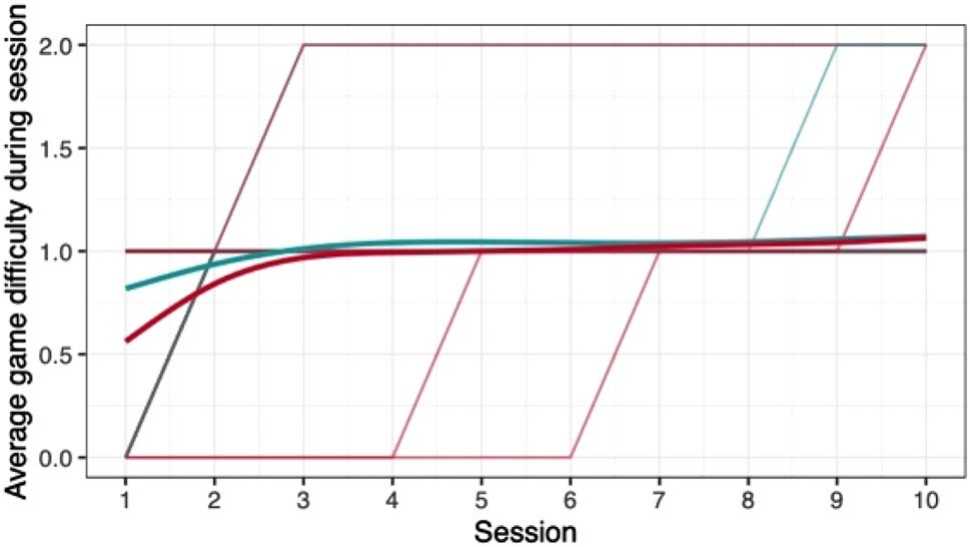
Alternative measure in word hunt

When comparing the means of game scores (mean pwMS, mean HC, rank biserial correlation coefficient), no clear differences between pwMS (*n = *30) and HC (*n = *29) were found: *Low Pop* (11.6, 11.9, |*r*_rb_|= 0.188), *Baggage Claim* (2.14, 2.51, |*r*_rb_|= 0.378), *Face Switch* (26.8, 28.5, |*r*_rb_|= 0.11), *Must Sort* (80.7, 85.7, |*r*_rb_|= 0.125), *Perilous Path* (6.8, 7.0, |*r*_rb_|= 0.053), *Puzzle Blox* (3.0, 3.2, |*r*_rb_|= 0.096), *Rush Back* (43.6, 47.6, |*r*_rb_|= 0.193), *Spin Cycle* (1.5, 1.7, |*r*_rb_|= 0.182), *Word Hunt* (2.3, 2.0, |*r*_rb_|= 0.255), *Zap Gap* (4.3, 4.8, |*r*_rb_|= 0.174). When the average scores of each session for each participant are plotted, an increase in game-scores over the 5-week study period is visible, with the control-group showing steeper increases in some, and higher scores and or difficulty levels reached in most games (Figs. 1, 2, 3, 4, 5, 6, 7, 8, 9, 10). The average score of all but two games (*Must Sort and Word Hunt)* showed a continuous increase over the ten sessions. When we plotted the data obtained from these two games using *mean difficulty level* instead of *number of successful rounds* and *completion time* as game measures, an increase of average performance was visible as well (Figs. 11 and 12).

#### Group differences in game acceptance ratings

There were no clear differences regarding acceptance ratings between pwMS and HC. An overview of the game acceptance ratings can be found in the supplementary material (Table S2: Mean acceptance ratings on Likert scale (1–5) by group and topic (HC vs pwMS)).

## Discussion

### Correlation with established neuropsychological assessments

All but one of the adaptive cognitive games showed a moderate–strong correlation with their respective pre-assigned established reference tests and met the predefined correlation level (*r*_*s*_ ≥ 0.3) supporting their utility as cognitive assessment tools. The fact that the language-game *Word Hunt* did not reach a correlation coefficient of *r*_*s*_ ≥ 0.3 with RWT may be the effect of different assessment approaches: whereas Word Hunt relies on visual detection of words in a letter grid, the *RWT* is a purely verbal semantic fluency test. The overall correlation matrix, that shows moderate correlations of *Word Hunt* with all other reference tests (range *r*_*s*_ = 0.36–0.69), most of which include visual tasks, supports this assumption. Similarly, the only other predefined game-test correlation below *r*_*s*_ = 0.4: *Zap Gap – Stroop*, might have also been affected by the issue of different assessment approaches: while *Stroop* is a purely verbal test, in *Zap Gap* small orbs have to be touched at the right moment. Ergo visual-timing and dexterity might have influenced the comparison. Of course, other games also include a dexterity aspect which does usually not play a role in most paper pencil tests. However, dexterity required in the other games are very basic (touching large buttons to respond by yes/no). When omitting these two tests due to this effect, the other 8/10 correlations, where assessment methods are more similar, are distributed with a rather small range (*r*_*s*_ = 0.44–0.77).

Amongst the established reference tests, the correlation of SDMTs with the other tests stood out. Probably this relates to the fact that SDMT targets processing speed most and games are time-limited and, therefore, dependent on cognitive speed. This interpretation is supported both by the factor analysis, where the SDMT showed equal loadings for two of the four factors, and by the literature where the value of the SDMT as a practicable overall measure of CI in MS, is increasingly recognized [31]. The fact that speed is one of the main measures in many neuropsychological tests and cognitive games might have contributed to the many moderate–strong correlations, we were able to observe. Considering that many cognitive functions are based on information processing, this seems likely. However, while speed certainly is a factor shared by many games and neuropsychological tests, it alone did not explain all of our results. The tests and their corresponding games: VLMT, ROCF, Baggage Claim, and Perilous Path all have no time-factor and did not differ from the speed-based tests or games in the correlation matrix.

The many moderate–strong correlations with not only the preassigned reference tests but between the vast majority of games and established cognitive tests, depicted by the correlation matrix (Table 5), suggests that neither the games nor the chosen established neuropsychological reference tests are highly specific for single defined cognitive domains. To further investigate domain-affinity, we conducted a factor analysis with all cognitive games and reference tests. By categorizing each game and test into the factor with the strongest loading, we distinguished the following four factors by cognitive functions: 1. mental speed; 2. memory; 3. visual perception/recognition, and 4. mental flexibility. Whereas this categorization fits most games and tests, only 3/10 of the preassigned pairs of games and tests matched the same factor. These results show that even though a certain domain affinity was observable, an exclusive domain-based categorization was not possible. The fact that not only the games but also the reference tests, which are designed to assess specific cognitive domains, did not show clear domain-specificity suggests that this is more likely a genuine effect of the interdependence of cognitive domains and their measures rather than an issue of the game design only. Both the investigated cognitive games and the established reference tests seem to cover a broader spectrum of interdependent cognitive domains [32]. Furthermore, both test and game results depend on the measurement method chosen (e.g. mental processing, mental flexibility, and inhibition are mostly measured by speed). The observed correlation might therefore—at least in part—reflect the shared assessment measure, rather than an overlap of the cognitive domains. In any case, the main aim of this study was not to prove high domain-specificity, but rather to investigate whether cognitive games can reliably measure levels of cognitive performance.

The improvements observed in the scores achieved in the games reflect the practice effect which occurs when repeating a similar task multiple times [33]. Only in the games *Must Sort* (processing speed), and *Word Hunt* (language), we did not observe a clear increase in performance. For these two games, the predefined measures were raw scores (number of correct answers and completion time). We assume that the scores of these games stagnated or declined because the level-, and therefore difficulty change interfered with the measurement of raw scores. As performance improved, the difficulty level of these adaptive games increased, leading to less increase or even relative decrease in the raw scores. When we measured the performance over time by the *difficulty level reached*, a continuous increase in performance was seen for these two games as well despite the lower number of possible levels (Figs. 11 and 12). That training effects were consistently depicted by the games indirectly supports the value of cognitive games in measuring changes in performance but underlines the need to control for practice effects when assessing disease evolution over time. This task is not trivial as it is further complicated by evidence supporting a relation of practice effects in cognitive testing with disability progression as well as brain volume loss in pwMS [34]. Our study was not powered to detect group differences between pwMS and HCs. It is, therefore, not surprising that no clear group differences were found. Nevertheless, HC scored higher across all games and showed mostly steeper improvements during the study.

Overall, our results regarding correlation with established tests and change over time support the potential of cognitive games as measures of cognitive function in pwMS and HC. Our findings are in accordance with those of studies investigating gamified digital cognitive assessment methods in elderly people with CI, in patients post-stroke and in healthy individuals [12, 13] and one study using the tablet-based game *EVO-Monitor* to distinguish pwMS with CI from pwMS without CI, and from HC [7].

### Acceptance by participants

Our results clearly show that the games were not only appealing, but they were also recognized as beneficial to the management of their disease by the participating pwMS. We suspect that the gamification factor plays a large role in the enjoyment and motivation to complete the cognitive games. This assumption is supported by the participant’s feedback provided in a semi-structured interview conducted at EoS. Similarly, studies by Cerrato et al. (2017) and Wiley et al. (2020) also describe advantages of gamification elements regarding motivation, positive affect, enjoyment, felt challenge, meaning, and even performance in cognitive tasks [16, 35]. Such features are key for the ascertainment of good adherence, a critical requirement for a long-term monitoring tool of a chronic disease.

## Limitations

Since this was a sub study of the dreaMS feasibility study, the sample size, schedule, and prevalence of cognitive impairment were not specifically set up for the assessment of cognitive games [19]. The wide range of age and disability grades allowed by the inclusion criteria may have contributed to higher variability of the results and thus have reduced the power of the analysis. Since we are primarily interested in the potential of adaptive cognitive games as a monitoring tool, our aim is to show changes within one individual, rather than to compare to a population. Therefore, the wide age range should not have a major impact. Our study was not designed to further characterize amount and time course of the practice effects observed in this study, a known impediment of use as a monitoring tool [33, 36, 37]. Approaches such as varying the frequency of testing, providing multiple versions of the same test/game, or having an intense practice period with the goal of reaching the ceiling of the learning curve, need to be evaluated in further studies. Another inherent limitation of our study is related to the performance-dependent adaptation of the difficulty-levels. To prevent floor- and ceiling effects and help to avoid boredom (too easy) or frustration (too difficult) adapting the difficulty level is an important motivator [38]. On the flip side, adaptive levels interfere with interpretation of raw scores as long as these are not weighted by difficulty level. Establishing well-defined difficulty-levels and weighting the scores obtained according to difficulty level is a necessary prerequisite for the use of adaptive games as measures of cognitive performance. There might have been a recruitment bias towards people with technical affinity which might have influenced general performance and acceptance ratings.

## Conclusion

In this feasibility study, we demonstrate that smartphone games can provide reliable measures of cognitive function both in pwMS and HC. Although most game-derived measures correlated with their established cognitive reference tests, domain-affinity needs to be further explored in larger and more diverse populations. Practice effects were clearly depicted in both HCs and pwMS, suggesting that cognitive game measurements are sensitive to change over time and learning curves have to be taken into account in data analysis. All participants found the games appealing and meaningful and were motivated to use such a monitoring tool on a regular basis for longer periods. Further studies with longer duration in larger populations are warranted to validate such cognitive games as monitoring tools of cognition in pwMS. While the novel method explored in this study focused on monitoring disease evolution in pwMS, we acknowledge the great potential it shows as a rehabilitation tool in both MS and other medical fields.

## Supplementary Information

Below is the link to the electronic supplementary material.

Supplementary file1 (PDF 144 KB)

Supplementary file2 (PDF 85 KB)

Supplementary file3 (PDF 115 KB)

Supplementary file4 (PDF 7147 KB)

Supplementary file5 (PDF 101 KB)

Supplementary file6 (PDF 118 KB)
