# Supplementary material for: Assessment of cognitive performance in multiple sclerosis using smartphone-based training games: a feasibility study
Source: J Neurol. 2023 Mar 23;270(7):3451–63. doi: 10.1007/s00415-023-11671-9 (PMC10267276; doi:10.1007/s00415-023-11671-9)

Supplementary Material Figures S1-S6:

Exemplary Screenshots of Peak Games

Figure S1: Puzzle Blox

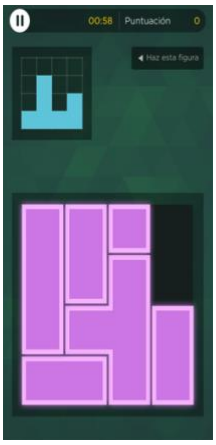

Figure S2: Must Sort

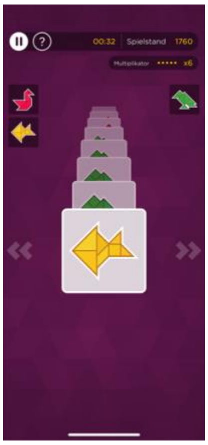

Figure S3: Face Switch

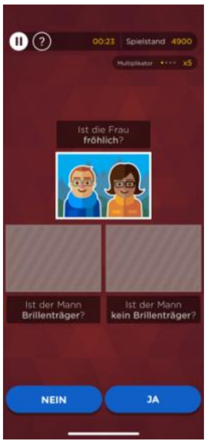

Figure S4: Low Pop

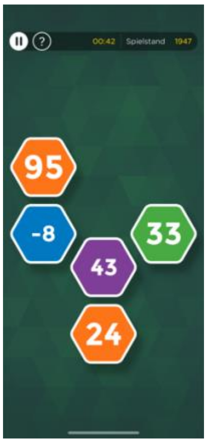

Figure S5: Word Hunt

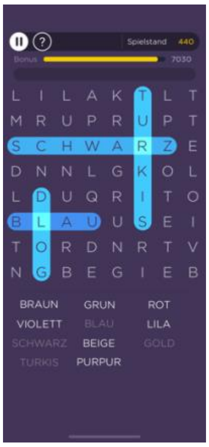

Figure S6: Spin Cycle

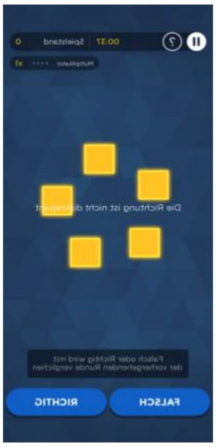

Supplement: Supplementary file 1 — Supplementary file1 (PDF 144 KB) [file 415_2023_11671_MOESM1_ESM.pdf]
