# Supplementary material for: Assessment of cognitive performance in multiple sclerosis using smartphone-based training games: a feasibility study
Source: J Neurol. 2023 Mar 23;270(7):3451–63. doi: 10.1007/s00415-023-11671-9 (PMC10267276; doi:10.1007/s00415-023-11671-9)
Supplement: Supplementary file 2 — Supplementary file2 (PDF 85 KB) [file 415_2023_11671_MOESM2_ESM.pdf]

# Supplementary Material S1:

Feedback questionnaire

| | Not at all | No | Partly | Yes | Absolutely |
|---|---|---|---|---|---|
| 1. Did you enjoy playing the game? | O | O | O | O | O |
| 2. Did you feel adequately challenged (not too easy and not too difficult) | O | O | O | O | O |
| 3. Would you be willing to play these games regularly in the future? | O | O | O | O | O |
| Question for pwMS only: | | | | | |
| 4. Did you think this game is relevant regarding MS? | O | O | O | O | O |
