# Supplementary material for: Assessment of cognitive performance in multiple sclerosis using smartphone-based training games: a feasibility study
Source: J Neurol. 2023 Mar 23;270(7):3451–63. doi: 10.1007/s00415-023-11671-9 (PMC10267276; doi:10.1007/s00415-023-11671-9)
Supplement: Supplementary file 3 — Supplementary file3 (PDF 115 KB) [file 415_2023_11671_MOESM3_ESM.pdf]

Supplementary Material S2:

Inclusion and exclusion criteria

Participants fulfilling all of the following inclusion criteria were eligible for the study:

A. Participants with MS

- Age 18-70
- Diagnosed with MS according to the revised McDonald criteria 2017, all clinical forms inclusive (CIS, RRMS, SPMS, PPMS)
- EDSS ≤ 6.5
- In possession of a dreaMS app compatible smartphone (iOS/Android, see section 3.2.4, Table 2)
- Corrected close visual acuity of ≥0.5
- Hand motor skills sufficient for using a smartphone
- Ability to follow the study procedures
- Informed Consent as documented by signature

B. Healthy controls (sex and age-matched to patients)

- Age 18-70
- In possession of a dreaMS app compatible smartphone (iOS/Android, see section 3.2.4, Table 2)
- Corrected close visual acuity of ≥0.5
- Hand motor skills sufficient for using a smartphone
- Being able to walk without aid
- Ability to follow the study procedures
- Informed Consent as documented by signature

The presence of any one of the following exclusion criteria lead to exclusion of the participant:

A. Participants with MS

- Other clinically significant concomitant disease states (e.g., renal failure, severe hepatic dysfunction, severe/unstable cardiovascular disease, progressive cancer, etc.)
- Known or suspected non-compliance, drug or alcohol abuse
- Women who are pregnant or breastfeeding
- Being a frequent PEAK* user (having used PEAK ≥ once daily over a period of ≥3 weeks) and not willing to abstain from using PEAK during the study period

B. Healthy controls (sex and age-matched to patients)

- Being diagnosed with MS or other disease affecting neurological and cognitive functions
- Other clinically significant concomitant disease states (e.g., renal failure, severe hepatic dysfunction, severe/unstable cardiovascular disease, progressive cancer, etc.)
- Known or suspected non-compliance, drug or alcohol abuse
- Women who are pregnant or breastfeeding
- Being a frequent PEAK* user (having used PEAK ≥ once daily over a period of ≥3 weeks) and not willing to abstain from using PEAK during the study period
