# Supplementary material for: Assessment of cognitive performance in multiple sclerosis using smartphone-based training games: a feasibility study
Source: J Neurol. 2023 Mar 23;270(7):3451–63. doi: 10.1007/s00415-023-11671-9 (PMC10267276; doi:10.1007/s00415-023-11671-9)
Supplement: Supplementary file 4 — Supplementary file4 (PDF 7147 KB) [file 415_2023_11671_MOESM4_ESM.pdf]

Clinical Study Protocol

DREAMS - Development of Digital Biomarkers in Multiple Sclerosis
Feasibility Study

A project funded by Innosuisse no. 33535.1 IP-ICT

Study Type:	Clinical trial with Medical Device (MD)
Study Categorisation:	Category C, Risk class I
Study Registration:	SNCTP Portal: to be named<br>ClinicalTrials.gov: NCT04413032
Study Identifier:	DreaMS_2020F
Sponsor, Sponsor-Investigator or Principal Investigator:	Prof. Ludwig Kappos<br>Department of Neurology University Hospital Basel and Research Centre for Clinical Neuroimmunology and Neuroscience Basel (RC2NB)<br>Petersgraben 4, 4031 Basel, Switzerland<br>Phone: +41 61 265.41.54<br>Fax: +41 61 265.41.98<br>*Ludwig.kappos@usb.ch*
Investigational Product:	DREAMS App (Medical Device Name)
Protocol Version and Date:	V1.2 Feasibility 17.09.2020

CONFIDENTIAL
The information contained in this document is confidential and the property of the Sponsor. The information may not - in full or in part - be transmitted, reproduced, published, or disclosed to others than the applicable Competent Ethics Committee(s) and Regulatory Authority(ies) without prior written authorisation from the Sponsor except to the extent necessary to obtain informed consent from those who will participate in the study.

Signature Page(s)

Study number	SNCTP Portal: to be named<br>ClinicalTrials.gov: NCT04413032
Study Title	DREAMS – Development of Digital Biomarkers in Multiple Sclerosis – Feasibility Study

The Sponsor-Investigator, his deputy and the Co-Investigator have approved the protocol version [V1.2 (dated 17.09.2020)], and confirm hereby to conduct the study according to the protocol, current version of the World Medical Association Declaration of Helsinki, ICH-GCP guidelines or ISO 14155 norm if applicable and the local legally applicable requirements.

Sponsor-Investigator:
Prof. Dr. med. Ludwig Kappos

Basel, 17.9.20 ______________________ L. Kappos ______________________
Place/Date Signature

Sponsor-Investigator Deputy:
Dr. med. Yvonne Naegelin

Basel, 17.9.2020 ______________________ [signature] ______________________
Place/Date Signature

Co-Investigator:
Dr. med. Johannes Lorscheider

Basel, 18.9.2020 ______________________ [signature] ______________________
Place/Date Signature

Statistician:
Andrea Wiencierz, PhD

Basel, 21.9.2020 ______________________ A. Wiencierz ______________________
Place/Date Signature

Local Principal Investigator at study site*:
I have read and understood this trial protocol and agree to conduct the trial as set out in this study protocol, the current version of the World Medical Association Declaration of Helsinki, ICH-GCP guidelines or ISO 14155 norm and the local legally applicable requirements.

Site	Department of Neurology<br>University Hospital Basel<br>and Research Centre for Clinical Neuroimmunology and Neuroscience Basel (RC2NB)<br>Petersgraben 4<br>4031 Basel<br>Switzerland
Principal Investigator	Prof. Dr. med. Ludwig Kappos

Basel, 17/9/2020
Place/Date

L. Kappos
Signature

Note: In multicentre studies, this page must be individually signed by all participating Local Principal Investigators.

Table of Contents

STUDY SYNOPSIS **7**
ABBREVIATIONS **13**
STUDY SCHEDULE TABLE 1 ..... **16**
1. STUDY ADMINISTRATIVE STRUCTURE **17**
1.1 Sponsor, Sponsor-Investigator 17
1.2 Principal Investigator(s) 17
1.3 Statistician ("Biostatistician") / Data Science 18
1.4 Data Administration 18
1.5 Monitoring institution 18
1.6 Data Safety Monitoring Committee 18
1.7 Any other relevant Committee, Person, Organisation, Institution 18
2. ETHICAL AND REGULATORY ASPECTS **19**
2.1 Study registration 19
2.2 Categorisation of study 19
2.3 Competent Ethics Committee (CEC) 19
2.4 Competent Authorities (CA) 19
2.5 Ethical Conduct of the Study 20
2.6 Declaration of interest 20
2.7 Patient Information and Informed Consent 20
2.8 Participant privacy and confidentiality 20
2.9 Early termination of the study 21
2.10 Protocol amendments 21
3. BACKGROUND AND RATIONALE **22**
3.1 Background and Rationale 22
3.2 Investigational Product (device) and Indication 24
3.2.1 Manufacturer 24
3.2.2 Summary 24
3.2.3 Components 24
3.2.4 Hardware 25
3.2.5 DREAMS Version used for Feasibility study 26
3.2.6 Data collected through the DREAMS App 28
3.2.7 Precautions for use 32
3.2.8 Software Safety Classification 32
3.2.9 User training 32
3.3 Preclinical Evidence 33
3.4 Clinical Evidence to Date 33
3.5 Medical Device: Rationale for the intended purpose in study (pre-market MD) 33
3.5.1 Introduction 33
3.6 Explanation for choice of comparator (or placebo) 35
3.7 Risks / Benefits 35
3.7.1 Risk analysis 35
3.7.2 Post-trial care 35
3.7.3 Competing studies 35
3.8 Justification of choice of study population 36
4. STUDY OBJECTIVES **36**
4.1 Overall Objective 36
4.2 Primary Objective 36
4.3 Exploratory Objectives 36
4.4 Safety Objectives 36

5. **STUDY OUTCOMES** 37
5.1 Primary Outcome 37
5.2 Exploratory Outcomes 37
5.3 Other Outcomes of Interest 37
5.4 Safety Outcomes 37
6. **STUDY DESIGN** 38
6.1 General study design and justification of design 38
6.2 Methods of minimising bias 38
6.2.1 Randomisation 38
6.2.2 Blinding procedures 38
6.2.3 Other methods of minimising bias 39
6.3 Unblinding Procedures (Code break) 39
7. **STUDY POPULATION** 40
7.1 Eligibility criteria 40
7.2 Recruitment and screening 41
7.3 Assignment to study groups 41
7.4 Criteria for withdrawal / discontinuation of participants 41
8. **STUDY INTERVENTION** 42
8.1 Identity of Investigational Products (treatment / medical device) 42
8.1.1 Experimental Intervention (treatment / medical device) 42
8.1.2 Control Intervention (standard/routine/comparator treatment / medical device) 44
8.1.3 Packaging, Labelling and Supply (re-supply) 44
8.1.4 Storage Conditions 45
8.2 Administration of experimental and control interventions 45
8.2.1 Experimental Intervention 45
8.2.2 Control Intervention 45
8.3 Dose / Device modifications 45
8.4 Compliance with study intervention 45
8.5 Technical Support 45
8.6 Data Collection and Follow-up for withdrawn participants 46
8.7 Trial specific preventive measures 46
8.8 Concomitant Interventions (treatments) 46
8.9 Study Drug / Medical Device Accountability 46
8.10 Return or Destruction of Study Drug / Medical Device 46
9. **STUDY ASSESSMENTS** 47
9.1 Study flow chart(s) / table of study procedures and assessments 47
9.2 Assessments of outcomes 51
9.2.1 Assessment of primary outcome 51
9.2.2 Assessment of exploratory outcomes 51
9.2.3 Assessment of other outcomes of interest 51
9.2.4 Assessment of safety outcomes 51
9.2.5 Assessments in participants who prematurely stop the study 51
9.3 Procedures at each visit 52
9.3.1 Split into subtitles by type of visit 52
10. **SAFETY** 53
10.1 Medical Device Category C studies 53
10.1.1 Foreseeable adverse events and anticipated adverse device effects 53
10.1.2 Definition and Assessment of (Serious) Adverse Events and other safety related events 54

10.1.3 Reporting of (Serious) Adverse Events and other safety related events 56
10.1.4 Follow up of (Serious) Adverse Events 57
11. STATISTICAL METHODS 57
11.1 Hypothesis 57
11.2 Determination of Sample Size 57
11.3 Statistical criteria of termination of trial 57
11.4 Planned Analyses 57
11.4.1 Datasets to be analysed, analysis populations 57
11.4.2 Primary Analysis 58
11.4.3 Exploratory Analyses 59
11.4.4 Interim analyses 59
11.4.5 Safety analysis 59
11.4.6 Deviation(s) from the original statistical plan 59
11.5 Handling of missing data and drop-outs 60
12. QUALITY ASSURANCE AND CONTROL 61
12.1 Data handling and record keeping / archiving 61
12.1.1 Case Report Forms 61
12.1.2 Specification of source documents 61
12.1.3 Record keeping / archiving 61
12.2 Data management 61
12.2.1 Data Management System 61
12.2.2 Data security, access and back-up 62
12.2.3 Analysis and archiving 63
12.2.4 Electronic and central data validation 63
12.3 Monitoring 63
12.4 Audits and Inspections 63
12.5 Confidentiality, Data Protection 63
12.6 Storage of biological material and related health data 63
13. PUBLICATION AND DISSEMINATION POLICY 64
14. FUNDING AND SUPPORT 65
14.1 Funding 65
14.2 Other Support 65
15. INSURANCE 65
16. REFERENCES 66
17. APPENDICES 68
17.1 IMP: IB or SPC 68
17.2 Medical Devices: IB (according to ISO 14155) 68
17.3 Medical Devices: Assurance of producer 68
17.4 Medical Devices: List of norms (vollständig eingehaltene, teilweise eingehaltene) 68
17.5 APP contents 69
17.5.1. Questionnaires (in german) 69
17.5.1.1. MSIS-29 69
17.5.1.2. MSWS-12 70
17.5.1.3. Fatigue-Skala 71
17.5.1.4. MS Symptom Tracker 72
17.5.1.5. Relapse Protocol 73
17.6 Risk Management 76

STUDY SYNOPSIS

**Sponsor / Sponsor-Investigator**	Prof. Dr. med. Ludwig Kappos
**Study Title:**	DREAMS – Development of Digital Biomarkers in Multiple Sclerosis<br>Feasibility Study
**Short Title / Study ID:**	DREAMS<br>DreaMS_2020F
**Protocol Version and Date:**	V1.2 Feasibility 17.09.2020
**Trial registration:**	SNCTP Portal: to be named<br>ClinicalTrials.gov: NCT04413032
**Study category and Rationale**	Medical Device Category C, Risk Class I<br>(No CE and not invasive)
**Clinical Phase:**	Phase of development (Feasibility)

**Background and Rationale:**	Multiple Sclerosis (MS) is a chronic inflammatory disease of the central nervous system (CNS) causing focal lesions of demyelination and diffuse neurodegeneration in the grey and white matter of the brain and spinal cord, leading to physical and cognitive disability. Currently there is a limited number of relevant biomarkers available in patients with MS, such as clinical, imaging or biological measures. Patient history and neurologic examination in combination with magnetic resonance imaging (MRI), evoked potentials and analysis of serum and cerebrospinal fluid (CSF) are the gold standard of diagnosis and mainly patient history, neurologic examination and MRI are used for patient monitoring. However, their prognostic value on a patient level is still very limited. Therefore, the scientific community and patients are in need for new and more reliable biomarkers, especially biomarkers of disease progression in order to adapt therapeutic approaches on an individual level. Digital biomarkers have the potential to fill this gap allowing for quasi-continuous measures that might be more informative than episodically collected conventional data concerning the impact of the disease on activities of daily living.<br>Together with Healios, a Basel-based company specialised in remote patient monitoring solutions we have developed the DREAMS App, a data collection, communication and management platform using data collected through the patients' mobile devices (smartphone and wearables). Using app-based challenges, continuous monitoring and surveys we aim to obtain data that can be used as digital biomarkers. These digital biomarkers will provide more granular and precise assessments, thus complementing traditional diagnostic measures and techniques. In this feasibility study a number of digital biomarkers will be applied to test their technical reproducibility/stability and meaningfulness in a group of patients with MS and healthy controls to select the most informative for the thereafter planned validation studies.
**Objective(s):**	The primary objective is to identify digital biomarkers that are technically reliable and measurable, and perceived as user friendly and meaningful for patients with MS (PwMS).<br>The exploratory objective of this study is to find differences on these biomarkers between 30 PwMS and 30 age- and sex-matched healthy controls (HC) on a group level.

**Outcome(s):**	The primary study outcomes are:<br>1. Test reliability of digital biomarkers measured by Intra Class Correlation (ICC≥0.6)<br>2. Test reliability of digital biomarkers measured by Coefficient of variation (CV< 20%)<br>3. Determination of user acceptance of digital biomarkers with regards to acceptance based on questionnaire (>3 on a Likert Scale)<br>Exploratory study outcome is to identify digital biomarkers that:<br>a.) differenciate on a group level between PwMS and age- and sex- matched healthy controls (HC).<br>b.) correlate with already established assessment tools in their respective domains (e.g. "Catch-a-cloud" vs. Nine-Hole-Peg test).
**Study design:**	This feasibility study is a prospective observational proof of concept study in patients with MS with a control group of healthy volunteers. Duration of the study will be 6 weeks per patient. This study is the first step to identify precise, well accepted and meaningful digital biomarkers for patients with multiple sclerosis as a first step towards validation in further studies. Digital biomarkers will be captured by patient's own smartphones (iOS and Android) and by an additional smartwatch (Fitbit Versa 2).<br>30 patients with MS and 30 age- and sex- matched healthy controls will be included. All participants will be instructed by a study nurse at the beginning of the study to assure that all patients and healthy controls will use the App and perform all tasks in a similar and correct way. Within the study all participants will perform all tests repeatedly over a time period of 5 weeks (every test twice a week resulting in 10 repetitive performances/test). In week number 6 they will perform all questionnaires and will have their semi-structured interview.<br>Technical reproducibility of the markers will be the main criterion for reliability (ICC≥0.6, CV<20, s. outcomes) and meaningfulness will be assessed by capturing expert and patient opinion, the latter obtained by a feedback questionnaire and a semi-structured interview at the end of the study.<br>Technical features and reproducibility of the tests will be also assessed in a group of healthy controls. In an exploratory analysis potential differences between healthy controls and PwMS on a group level will be assessed.

**Inclusion / Exclusion criteria:**	<u>Inclusion Criteria:</u><br>For Participants with MS<br>- Age 18-70<br>- Diagnosed with MS according to the revised McDonald criteria 2017, all clinical forms inclusive (CIS, RRMS, SPMS, PPMS)<br>- EDSS ≤ 6.5<br>- In possession of a DREAMS App compatible smartphone (iOS/Android)<br>- Corrected close visual acuity of ≥0.5<br>- Hand motor skills sufficient for using a smartphone<br>- Ability to follow the study procedures<br>- Informed Consent as documented by signature<br><br>For Healthy controls (sex and age matched to patients)<br>- Age 18-70<br>- In possession of a DREAMS App compatible smartphone (iOS/Android)<br>- Corrected close visual acuity of ≥0.5<br>- Hand motor skills sufficient for using a smartphone<br>- Being able to walk without aid<br>- Ability to follow the study procedures<br>- Informed Consent as documented by signature<br><br>Exclusion Criteria:<br>For Participants with MS<br>- Other clinically significant concomitant disease states (e.g., renal failure, severe hepatic dysfunction, severe/unstable cardiovascular disease, progressive cancer, etc.)<br>- Known or suspected non-compliance, drug or alcohol abuse<br>- Women who are pregnant or breast feeding<br>- Being a frequent PEAK*user (having used PEAK ≥ once daily over a period of ≥3 weeks) and not willing not to use PEAK during the study period<br>*PEAK: freely available App for cognitive training<br><br>For healthy controls (sex and age matched to patients)<br>- Being diagnosed with MS or other disease affecting neurological and cognitive functions<br>- Other clinically significant concomitant disease states (e.g., renal failure, severe hepatic dysfunction, severe/unstable cardiovascular disease, progressive cancer, etc.)<br>- Known or suspected non-compliance, drug or alcohol abuse<br>- Women who are pregnant or breast feeding

	- Being a frequent PEAK*user (having used PEAK ≥ once daily over a period of ≥3 weeks) and not willing not to use PEAK during the study period<br>*PEAK: freely available App for cognitive training
**Measurements and procedures:**	DREAMS is a mobile application that is able to measure various digital biomarkers designed for this study. More than 20 digital biomarkers (visual, motoric (active/passive), cognitive) will be tested along a defined schedule. Technical reproducibility of the markers and the meaningfulness for patients will be the main criteria for selection to the following validation study.
**Study Product / Intervention:**	Product: The DREAMS App<br>Intervention: During the study, users are requested to perform a series of tasks ("challenges"). During the challenges, the smartphone's sensors will gather data that can be analysed to measure the participants' performance and provide an indication of the level of impairment.
**Control Intervention (if applicable):**	Not applicable
**Number of Participants with Rationale:**	The sample size of 30 PwMS and 30 HC was estimated based on published results of similar research in the field. This number of participants is required to obtain sufficient data for the analysis (ICC, CV) of each potential biomarker.
**Study Duration:**	6 weeks per participant
**Study Schedule:**	Month Year of First-Participant-In (planned): 08/2020<br>Month Year of Last-Participant-Out (planned): 12/2020

**Investigator(s):**	Sponsor and Investigator:<br>Prof. Dr. med. Ludwig Kappos<br>Department of Neurology University Hospital Basel<br>and Research Centre for Clinical Neuroimmunology and Neuroscience Basel (RC2NB)<br>Petersgraben 4, 4031 Basel, Switzerland<br>Phone: +41 61 265.41.54<br>Fax: +41 61 265.41.98<br>*Ludwig.kappos@usb.ch*<br>Deputy and Co-PI:<br>Dr. med. Yvonne Naegelin<br>Department of Neurology University Hospital Basel<br>and Research Centre for Clinical Neuroimmunology and Neuroscience Basel (RC2NB)<br>Petersgraben 4, 4031 Basel, Switzerland<br>Phone: +41 61 265.41.51<br>Fax: +41 61 265.41.00<br>Yvonne.naegelin@usb.ch<br>Co-PI:<br>Dr. med. Johannes Lorscheider<br>Department of Neurology University Hospital Basel<br>and Research Centre for Clinical Neuroimmunology and Neuroscience Basel (RC2NB)<br>Petersgraben 4, 4031 Basel, Switzerland<br>Phone: +41 61 265.41.51<br>Fax: +41 61 265.41.00<br>Johannes.lorscheider@usb.ch
**Study Centre(s):**	Single-centre study at the Department of Neurology (MS Centre), University Hospital Basel, Switzerland.
**Role of the external cooperation partner for technical development: HEALIOS GmbH Sevogelstrasse 32 4052 Basel, Switzerland**	Together with Healios, a Basel-based company specialised in remote patient monitoring solutions we have developed the DREAMS App, a data collection, communication and management platform using data collected through the patients' mobile devices (smartphone and wearables). This project is funded by Innosuisse – Swiss Innovation Agency (Funding Agreement Innovation project 33535.1 IP-ICT).<br>RC2NB and Healios GmbH have agreed (and signed a cooperation agreement) on cooperation partnership for the duration of 6 years to build and continuously develop the "MS digital solution" (explained in the protocol, section 3.2.). The DREAMS App is part of this development. The agreement defines the terms concerning Deliverables, Financial Terms, Intellectual Property Rights, Exploitation Rights, Data Governance.

**Statistical Considerations:**	The sample size was estimated based on published results of similar research in the field (Midaglia et al., J Med Internet Res. 2019). All subjects who complete the study will be included in the primary analysis (per protocol analysis).<br>Primary analyses:<br>The active tests will be selected using a stepwise selection strategy. In step 1, the test reliability will be examined with intra-class-correlation (ICC) and median coefficient of variance (CV). In step 2, user acceptance and adherence will be determined: Feedback on user acceptance will be captured with a feedback questionnaire and a semi-structured interview in week 6. Adherence will be calculated as percentage of completed repetitions divided by the total number of scheduled repetitions for each test. In step 3, all tests that fulfil the minimal requirements for reliability in either ICC or CV, as well as user acceptance and adherence, will be ranked according to their performance in steps 1 and 2. Based on this ranking, tests for each domain will be selected to be further analysed in the validation study.<br>Exploratory Analyses:<br>Potential differences between healthy controls and PwMS on a group level will be analysed with appropriate statistical tests, depending on data category and distribution (e.g. Wilcoxon Signed Rank Test). Although the study is not designed to detect statistically significant differences, results may be used in order to inform test selection for the validation study. For vision tests, dexterity and cognitive tests, correlations with established tests (e.g. "Catch-a-cloud" vs. Nine-Hole-Peg test) will be analysed in order to inform test selection for the validation study.
**GCP Statement:**	This study will be conducted in compliance with the protocol, the current version of the Declaration of Helsinki, the ICH-GCP or ISO EN 14155 (as far as applicable) as well as all national legal and regulatory requirements.

ABBREVIATIONS

ADEM	Acute Disseminated Encephalomyelitis
AE	Adverse Event
App	Application Software
AWS	Amazone Web Services
BASEC	Business Administration System for Ethical Committees, (https://submissions.swissethics.ch/en/)
BL	Baseline
BMI	Body Mass Index
CA	Competent Authority (e.g. Swissmedic)
CEC	Competent Ethics Committee

CE Mark	CE marking is a certification mark that indicates conformity with health, safety, and environmental protection standards for products sold within the European Economic Area
CIS	Clinically Isolated Syndrome
CNS	Central Nervous System
CSF	Cerebrospinal Fluid
CRF	Case Report Form
ClinO	Ordinance on Clinical Trials in Human Research *(in German: KlinV, in French: OClin, in Italian: OSRUm)*
eCRF	Electronic Case Report Form
CTCAE	Common terminology criteria for adverse events
CV	Coefficient of Variance
dBM	Digital Biomarker
DMT	Disease Modifying Treament
DOB	Date of Birth
DSUR	Development safety update report
EMS	Exploration Module for Multiple Sclerosis
EDSS	Expanded Disability Status Scale
GCP	Good Clinical Practice
HC	Healthy Controls/Healthy Control Persons
IB	Investigator's Brochure
ICC	Intra-Class-Correlation
HADS	Hospital Anxiety and Depression Scale
Ho	Null hypothesis
H1	Alternative hypothesis
HRA	Federal Act on Research involving Human Beings *(in German: HFG, in French: LRH, in Italian: LRUm)*
IMP	Investigational Medicinal Product
IIT	Investigator-initiated Trial
ISO	International Organisation for Standardisation
ITT	Intention to treat
MD	Medical Device (in the case of DREAMS, it is a Software as a Medical Device (SaMD)
MedDO	Medical Device Ordinance *(in German: MepV, in French: ODim)*
MS	Multiple Sclerosis
MSFC	Multiple Sclerosis Functional Composite
MRI	Magnetic Resonance Imaging
MUSIC	Multiple Sclerosis Inventory of Cognition
NMO	Neuromyelitis Optica
ON	Optic Neuritis
PASAT	Paced Auditory Serial Addition Test
PI	Principal Investigator
PPMS	Primary Progressive Multiple Sclerosis
PwMS	Patient with Multiple Sclerosis
ROCF	Rey-Osterrieth Complex Figure Test
RRMS	Relapsing-Remitting Multiple Sclerosis
S	Screening
SaMD	Software as Medical Device
SDV	Source Data Verification

SDMT	Symbol Digit Modalities Test
SOP	Standard Operating Procedure
STROOP	Colour-Word-Interference Test
SPC	Summary of product characteristics
SPMS	Secondary Progressive Multiple Sclerosis
SUSAR	Suspected Unexpected Serious Adverse Reaction
T25FW	Timed 25-Follt Walk Test
TMF	Trial Master File
TMT	Trail Making Test
V	Visit
VLMT	Verbal Learn and Memory Test
VPN	Virtual Private Network
9-HPT	9-Hole-Peg-Test

STUDY SCHEDULE TABLE 1

Table 1

		Week -1	Week 1	Week 2	Week 3	Week 4	Week 5	Week 6	
Visit Name	S	BL							End of Study
Visit Number	V1	V2							V3
Participant Information and Informed Consent	+								
In- /Exclusion Criteria	+								
Medical* History	+								
App download and test instructions		+							
Vision tests (contrast vision inclusive)		+							
EDSS* (PwMS only)		+							+
MSFC, SDMT*		+							
Neuro-psychological Testing		+							
Test performed by study participants (App active and passive monitoring)			+	+	+	+	+		
Questionnaires								+	
Feedback Questionnaire and Semi-Structured Interview									+

Table 1: Overall Study Schedule (Feasibility)

S=Screening, BL=Baseline, * Data will be taken of routine visit if this visit is +/- 2 weeks around BL. If this is not feasible data will be collected the latest at BL.
MSFC= Multiple Sclerosis Functional Composite (T25FW, 9-HPT), SDMT= Symbol Digit Modalities Test

1. STUDY ADMINISTRATIVE STRUCTURE

1.1 Sponsor, Sponsor-Investigator

Prof. Dr. med. Ludwig Kappos
Department of Neurology University Hospital Basel
and Research Centre for Clinical Neuroimmunology and Neuroscience Basel (RC2NB)
Petersgraben 4, 4031 Basel, Switzerland
Phone: +41 61 265.41.54
Fax: +41 61 265.41.98
Ludwig.kappos@usb.ch

Prof. Ludwig Kappos is the Sponsor and Principal Investigator of this study: Supervision Co-PI's are Dr. Yvonne Naegelin and Dr. Johannes Lorscheider: study design, staffing, collection, management, analysis and interpretation of data as well as writing of the report.

1.2 Principal Investigator(s)

Prof. Dr. med. Ludwig Kappos
Department of Neurology University Hospital Basel
Petergraben 4, 4031 Basel, Switzerland
Phone: +41 61 265.41.54
Fax: +41 61 265.41.98
Ludwig.kappos@usb.ch

Prof. Ludwig Kappos is the Sponsor and Principal Investigator of this study.
He is supervising his Co-PIs Dr. Yvonne Naegelin and Dr. Johannes Lorscheider concerning study design, staffing, collection, management, analysis and interpretation of data as well as writing of the report.

Deputy and Co-PI:
Dr. med. Yvonne Naegelin
Department of Neurology University Hospital Basel
Petergraben 4, 4031 Basel, Switzerland
Phone: +41 61 265.41.51
Fax: +41 61 265.41.00
Yvonne.naegelin@usb.ch

Co-PI:
Dr. med. Johannes Lorscheider
Department of Neurology University Hospital Basel
Petergraben 4, 4031 Basel, Switzerland
Phone: +41 61 265.41.51
Fax: +41 61 265.41.00
Johannes.lorscheider@usb.ch

1.3 Statistician ("Biostatistician") / Data Science

Andrea Wiencierz, PhD
Senior statistician
Clinical Trial Unit Basel (CTU)
Departement Klinische Forschung University of Basel
Spitalstrasse 12,

4031 Basel, Switzerland
Phone: +41 61 328.77.17
Andrea.Wiencierz@usb.ch

1.4 Data Administration

Bram Stieltjes, MD, PhD
Department of Radiology
University Hospital Basel
Petersgraben 4

4031 Basel, Switzerland
Phone: +41 61 328.51.75
Bram.stieltjes@usb.ch

1.5 Monitoring institution

Clinical Trial Unit Basel (CTU)
Departement Klinische Forschung University of Basel
Spitalstrasse 12,

4031 Basel, Switzerland
Phone: +41 61 328.77.17

1.6 Data Safety Monitoring Committee

DSMC is not needed as this is not an interventional study.

1.7 Any other relevant Committee, Person, Organisation, Institution

External cooperation partner for technical development:

Guilhem Dupont
President & CEO
HEALIOS GmbH
Sevogelstrasse 32

4052 Basel, Switzerland
Phone: +41 79 295.92.89
Guilhem.dupont@healios.io

2. ETHICAL AND REGULATORY ASPECTS

The decision of the CEC and Swissmedic/foreign competent authority concerning the conduct of the study will be made in writing to the Sponsor-Investigator before commencement of this study. The clinical study can only begin once approval from all required authorities has been received. Any additional requirements imposed by the authorities shall be implemented.

2.1 Study registration

SNCTP Portal: to be named
ClinicalTrials.gov: NCT04413032, Registration Date: 02.06.2020

2.2 Categorisation of study

The Medical Device (Software as medical device, SaMD) of this study falls under Category C (risk class I) as it is not authorised in Switzerland, and it does not have a conformity marking. The MD is not invasive.

2.3 Competent Ethics Committee (CEC)

We ensure that approval from an appropriately constituted Competent Ethics Committee (CEC) is sought for the clinical study:
Ethikkommission Nordwest- und Zentralschweiz (EKNZ)
Hebelstrasse 53 – 4056 Basel, Switzerland
We will adhere to all reporting duties and allowed time frame (all changes in the research activity and all unanticipated problems involving risks to humans; including in case of planned or premature study end and the final report) and no changes are made to the protocol without prior Sponsor and CEC approval.
Premature study end or interruption of the study is reported within 15 days. The regular end of the study is reported to the CEC within 90 days, the final study report shall be submitted within one year after study end. Amendments are reported according to chapter 2.10.

2.4 Competent Authorities (CA)

We will obtain approval from the competent authority (Swissmedic) before the start of the clinical trial.
We will adhere to all reporting duties and allowed time frame to CA including the reporting duties in case of planned or premature study end and the final report. Reporting duties and timelines are the same as for CEC, except of non-substantial amendments that shall be reported as soon as possible. Amendments are reported according to chapter 2.10.

2.5 Ethical Conduct of the Study

The study will be carried out in accordance to the protocol and with principles enunciated in the current version of the Declaration of Helsinki, the guidelines of Good Clinical Practice (GCP) issued by ICH, in case of medical device: the European Regulation on medical devices 2017/745 and the ISO Norm 14155 and ISO 14971, the Swiss Law and Swiss regulatory authority's requirements. The CEC and regulatory authorities will receive annual safety and interim reports and be informed about study stop/end in agreement with local requirements.

2.6 Declaration of interest

The Sponsor-Principal Investigator Prof. Ludwig Kappos and his Co-PI's Dr. med. Yvonne Naegelin and Dr. med. Johannes Lorscheider have no conflict of interest (independence, intellectual, financial, proprietary etc.).

2.7 Patient Information and Informed Consent

All participants will be informed orally and in written format (ICF) about the study by Dr. Yvonne Naegelin, Dr. Johannes Lorscheider or the responsible Study Physician / Study Nurse. Consent is sought from each participant.
As a compensation for participating, every participant can keep his/her smartwatch Fitbit versa2 device after the end of the study. Upon completion of the study the App will no longer be accessible for the study participants.
The investigators will explain to each participant the nature of the study, its purpose, the procedures involved, the expected duration, the potential risks and benefits and any discomfort it may entail. Each participant will be informed that the participation in the study is voluntary and that he/she may withdraw from the study at any time and that withdrawal of consent will not affect his/her subsequent medical assistance and treatment.
The participant must be informed that his/her medical records may be examined by authorised individuals other than their treating physician.
All participants of the study will be provided a participant information sheet and a consent form describing the study and providing sufficient information for participants to make an informed decision about their participation in the study. Every participant will be given at least 24 hours time to decide whether to participate or not.
The formal consent of a participant, using the approved consent form, must be obtained before the participant is submitted to any study procedure.
The participant should read and consider the statement before signing and dating the informed consent form, and should be given a copy of the signed document. The consent form must also be signed and dated by the investigator (or his designee) at the same time as the participant sign, and it will be retained as part of the study records.

2.8 Participant privacy and confidentiality

The investigator affirms and upholds the principle of the participant's right to privacy and that they shall comply with applicable privacy laws. Especially, anonymity of the

participants shall be guaranteed when presenting the data at scientific meetings or publishing them in scientific journals.
Individual subject medical information obtained as a result of this study is considered confidential and disclosure to third parties is prohibited. Subject confidentiality will be further ensured by utilising subject identification code numbers to correspond to data in the computer files.
For data verification purposes, authorised representatives of the Sponsor (-Investigator), a competent authority (e.g. Swissmedic), or an ethics committee may require direct access to parts of the medical records relevant to the study, including participants' medical history.
For the App to function well, the participant will be requested to allow the DREAMS App's access to items such as:

- Motion & fitness (for phone movement tracking)
- Microphone (for speech recognition)
- Camera (to measure distance between eyes and screen)
- HealthKit or GoogleFit (for access to data made available by Apple and Google), this is OPTIONAL (those data would be used for verification of data derived from DREAMS)
- Notifications and reminders (to help remind the participant when to perform the activities)

2.9 Early termination of the study

The Sponsor-Investigator (and any competent authority) may terminate the study prematurely according to certain circumstances, for example:

- ethical concerns,
- insufficient participant recruitment,
- when the safety of the participants is doubtful or at risk, respectively,
- alterations in accepted clinical practice that make the continuation of a clinical trial unwise,
- early evidence of benefit or harm of the experimental intervention

2.10 Protocol amendments

The Sponsor-Principal Investigator Prof. Ludwig Kappos and his Co-PI's Dr. med. Yvonne Naegelin and Dr. med. Johannes Lorscheider are allowed to amend the protocol or to provide suggestions for a protocol amendment.
Important protocol modifications (e.g., changes to eligibility criteria, outcomes, analyses) will be communicated within two weeks to relevant parties (e.g., investigators, CEC, competent authorities, trial participants, trial registries, journals, regulators).
Substantial amendments are only implemented after approval of the CEC and CA respectively.
Under emergency circumstances, deviations from the protocol to protect the rights, safety and well-being of human subjects may proceed without prior approval of the sponsor and the CEC/CA. Such deviations shall be documented and reported to the sponsor and the CEC/CA as soon as possible.
All non-substantial amendments are communicated to the CA as soon as possible if applicable and to the CEC within the Annual Safety Report (ASR).

3. BACKGROUND AND RATIONALE

3.1 Background and Rationale

Multiple Sclerosis (MS) is a chronic inflammatory disease of the central nervous system (CNS) causing focal lesions of demyelination and diffuse neurodegeneration in the grey and white matter of the brain and spinal cord, leading to physical and cognitive disability[14]. A majority of patients primarily presents with a relapsing remitting course of disease (RRMS), followed by a progressive phase (secondary progressive MS, SPMS) [15]. Currently there is a limited number of relevant biomarkers available in patients with MS, such as clinical, imaging or biological measures [16]. Patient history and neurologic examination in combination with magnetic resonance imaging (MRI), evoked potentials and analysis of serum and cerebrospinal fluid (CSF) are the gold standard of diagnosis and mainly patient history, neurologic examination and MRI are used for patient monitoring. However, their prognostic value on a patient level is still very limited. Therefore, the scientific community and patients are in need for new and more reliable biomarkers, especially biomarkers of disease progression in order to adapt therapeutic approaches on an individual level. Digital biomarkers have the potential to fill this gap allowing for quasi continuous assessment that should be more informative than episodically collected conventional data especially concerning the impact of the disease on activities of daily living. Digital biomarkers have the potential to be even more sensitive to changes than conventional biomarkers have been so far.

Digital biomarkers are defined as objective, quantifiable physiological and behavioural data that are collected and measured by means of digital devices such as portables, wearables, implantables or digestibles. An overview over the use of mobile devices to measure outcome in clinical research has been published [17]. It is important to mention that the idea of a digital biomarker is not to replicate what is done in clinical practice but is to generate a novel measure of the disease itself. It is the common understanding, that a biomarker has to be validated first before its clinical use.

There are some groups working on digital biomarkers for multiple sclerosis. The main three groups are 1. "Floodlight" from Roche Pharma AG, for open use as "Floodlight open" App, 2. "myMS" from the group of Prof. Daniel Pelletier from the university of southern California and 3. "MSCopilot" from a group of French neurologists. The Apps of working groups 1 and 2 have no validated digital biomarkers yet. Roche Pharma AG is concentrating on the use of the App with its current non validated biomarkers, while the group of Daniel Pelletier is trying to collect data of 200 patients with MS that will be compared to conventional biomarkers to validate his tool. 3. "MSCopilot" consisting of 4 tests (walking, dexterity, cognition (SDMT) and vision) was assessed within a clinical study and reliability was confirmed when tested against the standard "Multiple Sclerosis Functional Composite (MSFC)" [18].

Our own developed DREAMS App is a data collection, communication and management platform collecting data from mobile devices (smartphone and wearables) owned by the patient. Through the use of App-based tests, surveys and sensor data, we aim to identify

novel types of clinical data that can be used as digital biomarkers with complementary clinical value as compared to the traditional diagnostic methods and techniques.

In this first study with the DREAMS App we plan to prove technical feasibility and meaningfulness to patients with MS. In a second study (not part of this protocol) we will prospectively validate those digital biomarkers that proved to be technically reliable and measurable as well as meaningful for patients during this first study in two well defined prospectively followed patient cohorts (SMSC, GeneMSA/SUMMIT). We do not only aim at identifying single biomarkers that correlate with conventional measures but also aim at deriving patterns of biomarkers to better characterise disease progression.

Our solution has a number of advantages compared to other existing tools: Floodlight has developed a battery of motor tests, but does not assess cognition comprehensively and does not include tests of visual acuity/contrast vision or questionnaires for important patient symptoms like fatigue. DREAMS includes more motor tests which matter to patients like climbing stairs and incorporates games that can used to assess the most important neuropsychological domains in a playful way.

To summarize our advantages, we developed more than 20 candidate digital biomarkers (e.g. measurement of speed or balance of walking) to be tested in a first approach (patients with MS versus healthy controls) to select the ones that are technically reliable and measurable as well as meaningful to our patients to later proceed with a selection of those biomarkers to validation (not part of this protocol). The App is planned to be a certified Software as Medical Device our development partner in order to be used by individuals with MS for self-monitoring, by health care providers in a clinical setting to improve clinical decision making and by clinical researchers as an outcome measure for clinical trials.

3.2 Investigational Product (device) and Indication

3.2.1 Manufacturer

The DREAMS App is developed together with Healios GmbH, with registered offices at Sevogelstrasse 32, CH-4052, Basel.

3.2.2 Summary

The DREAMS App, is intended to monitor the development of symptoms of PwMS over time by tracking disease relevant biomarkers. Monitoring is performed through a mobile application on the participant's smartphone (iOS or Android). The data generated using inbuilt sensors of the smartphone and a smartwatch are pseudonymized and transferred for storage and further processing and analysis in a secure central data unit.
The DREAMS App is a suite of digital tests to be performed using mobile phones or other electronic sensors, typically outside the clinic, during activities of daily living or in specific tasks via a Software as Medical Device (SaMD). The data generated by such digital tests are transformed by algorithms into metrics ("Test parameters") to characterize and monitor the MS disease in clinical practice and clinical development. After validation in further studies such Test parameters would also qualify for submission to regulatory authorities as approved endpoints ("Endpoints").

3.2.3 Components

DREAMS is a mobile application for PwMS, a central data processing unit in a secure computer environment, and a study portal to manage the studies via a web-browser.

Graph 2

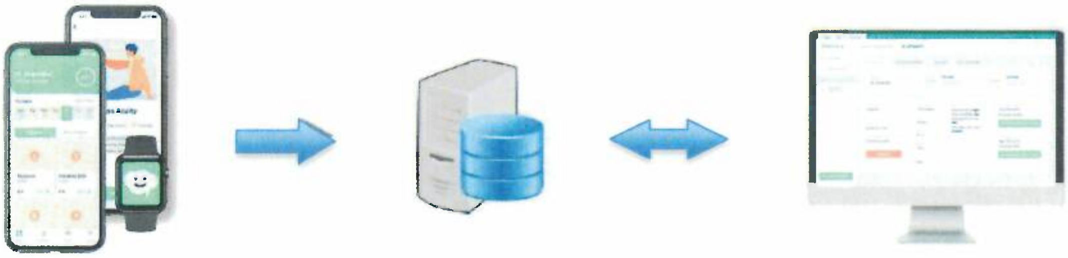

Graph 2: DREAMS mobile application

- **App** assesses (see details in Table 3):
  - Physical abilities via active and passive monitoring
  - Cognitive functions
  - Psychological impact through Patient Reported Outcome (Questionnaires)
  - Symptom Tracker
  - Relapse Reporting
- **Central database and processing unit** captures, stores and processes pseudonymized data.
- **Web portal** supports the study team with the administration and monitoring of study participants

3.2.4 Hardware

- The participants will use their own smartphone (iOS or Android).
- The DREAMS App is compatible with the following devices (iOS and Android):

Table 2

iOS	Android
• iPhone 6 and later<br>• iOS 11 or later<br>• 1GB RAM (automatically covered by range above)<br>• 500MB storage capacity available	• OS version 5.0 (API 21) or later<br>• Devices with GooglePlay services (some Chinese models don't have this)<br>• 2GB RAM<br>• 500MB storage capacity available<br>• Sensors: GPS, Accelerometer, Gyroscope, Magnetometer, Step Detector, Front Camera<br>• Typical brands and models/series that meet above requirements:<br>○ Samsung Galaxy S-series<br>○ Samsung Galaxy Notes-series<br>○ Samsung Galaxy A-series<br>○ Google Pixel series<br>○ Xiaomi Mi<br>○ Xiaomi Redmi<br>○ Huawei Y-series<br>○ Huawei P-series (lite)

Table 2: Compatible Smartphones

- The study nurse will install the DREAMS App from the app stores of Apple (App Store) and Android (GooglePlay) on participants smartphones and set up a study account.

- FitBit Versa2, provided by study team:
    - The Fitbit is a fitness tracker through which data about participant's heart rate, level of activity and sleep can be obtained.
    - The Fitbit Versa2 has a CE mark but is not a medical device in itself.
- Central database and processing unit
    - Central processing unit that manages the other 2 components (mobile App and study portal) as well as the data transfer from App and FitBit to the database.
    - Databases where the captured data will be stored securely and in a structured fashion.
    - To host the central database and processing unit, Healios has set up a virtual private network at Amazon WebServices. The dreaMS cloud is internally organized by a multitude of microservices in a "virtual private cloud" (VPC) hosted on a server farm by Amazon Web Services (AWS) in Frankfurt, Germany as primary zone. Dublin, Ireland is set-up as secondary zone with the objective of achieving high availability.
- Web browser
    - The study portal runs on any of the common web browsers (Chrome, Safari, Firefox, Microsoft Edge). Access to internet is required but no special software installations are needed.

3.2.5 DREAMS Version used for Feasibility study

For this study (Feasibility) the DREAMS App release R1.4 will be used. This release is including to following basic features:

a. **Mobile app**
    1. User authentication, schedule and reminders for the patients, available in German, English and Spanish
    2. Domains and challenges included (challenges names are listed as they will appear in the german version, table 3):

Table 3

Domain	Challenges & Scales
Movements	La Promenade<br>Pinguin<br>The Butler<br>Musical Chairs<br>Spanische Treppe<br>Schweizergarde
Fine motor skills	Catch-A-Cloud<br>Konfetti<br>Flame dodge (Left)<br>Flame dodge (Right)
Vision	Adlerauge<br>Nebel

Cognition	SDMT<br>Word Hunt<br>Spin Cycle<br>Low Pop<br>Puzzle Blox<br>Perilous Path<br>Baggage Claim<br>Must Sort<br>Rush Back<br>Face Switch<br>Zap Gap
Validated scales and Symptom Tracker	MSIS-29<br>MSWS-12<br>Fatigue Severity Scale<br>MS symptom Tracker<br>Feedback questionnaire
Relapse report	Relapse reporting
Passive monitoring	FitBit

Table 3: Domains and challenges included

b. **Web portal**
   1. The web portal supports the study nurse in administering the study and monitoring progress and adherence of participants.
   2. Main features available in R1.4 are:
      - Multiple studies
      - 3 languages
      - New Participant Introduction tailored to each study
      - Participant management
      - Relapse reporting
      - Schedule monitoring
      - Nurse and patient support

c. **Back-end**
   1. AWS infrastructure for communication and data capture
   2. Algorithms to extract test features from each of the challenges

3.2.6 Data collected through the DREAMS App

In addition to the results of all challenges, the following data are being collected through the DREAMS App:

Table 4

**Data Classification**		**Data Items**	**Explanation/Comments**
**1. Subject Data**			
	1.1. Device Data		
		IP address	IP (Internet protocol) address of the smartphone
		Device model	Device make (manufacturer, e.g. Apple/Samsung/...) and model (e.g. iPhone XS)
		Device OS	Device OS version
		App version	DREAMS app version
		Language	User's language setting
		Access to Google Services	Approval of access to Google Services
		Access to required sensors	Approval of access to sensor data which is captured while the user if performing challenges with the DREAMS App.<br>Raw sensor data is collected while the user is performing the challenges. This raw data is cleaned-up from noise and analysed in order to extract the different metrics that can be interpreted by and provide more clinical insight to the doctor.
	1.2. DREAMS Account		
		Avatar chosen	Avatar selected by user
		Nickname chosen	Nickname set by user
	1.3. DREAMS analytics		
		Access log	Date and time of using the app
		Time using the App	Duration of app activation and usage
		Screens visited	Screens of DREAMS app visited
		Crashlytics (bug tracker)	App service to report in case the app crashes and registers and transmits technical data to support the technical team to

			identify root causes to prevent repetition
	1.4. Personal information (This information will be collected at study baseline visit by the Study Nurse)		
		Telephone number	Authentication and verification of the uses personally identifiable information (PII). Healios does not have access to data that directly identifies the user.
		Year of Birth	Year of Birth (YYYY)
		Height (optional)	Height (in cm)
		Weight (optional)	Weight (in kg)
		Gender (optional)	Male/Female
		Participant type (PwMS/HC) (optional)	Patient with MS/Healthy participant
		Vision acuity >50%?	Yes/No
		User wears glasses or lenses?	Yes/no
		Was user able to perform Flame Dodge	Yes/No
		Mobile platform	iOS/Android
		Smartphone model	Is smartphone model supported by DREAMS? Yes/No
		Does participant have balance problem?	Yes/No
		Does participant use walking aid?	No assistive device<br>AFO (Ankle-Foot Orthosis)<br>Electrical stimulation device<br>Can/crutch – unilateral<br>Can/crutch – bilateral<br>Rolling walker<br>Non-rolling walker
	1.5 Connected apps and devices		
		Study ID for Fitbit and PEAK app	De-identified user account
		Password for Fitbit and PEAK app	Password
**2. Passive activity data from smartphone**			
	2.1. iOS device (HealthKit)		This data is collected in the background through iOS' HealthKit service upon approval from the participant (optional along ICF)
		Basal energy burned	Resting energy is the energy that the user's body burns to maintain its normal, resting state. The body uses this energy to perform basic functions like

			breathing, circulating blood, and managing the growth and maintenance of cells.
		Active energy burned	Active energy is the energy that the user has burned due to physical activity and exercise.
		Heart rate	User's heart rate if user has a heart rate monitor connected to HealthKit
		Heart rate variability SDNN	Standard deviation of heartbeat intervals.
		Resting heart rate	Heart rate when user's body is in normal, resting state.
		Walking heart rate	Heart rate when user's body is active due to physical activity and exercise.
		Distance walking /running	Distance user walks/runs
		Apple exercise time	Amount of time HealthKit registered the user has performed physical activity and exercise.
		Step count	Number of steps counted.
		Flights climbed	Measures the number flights of stairs that the user has climbed as measured by Healthkit and/or connected apps.
		Sleep analysis*	Sleep analysis performed by iOS if data is provided by user through manual intervention or connected app or device.
	2.2. Android device (Google Fit)		This data is collected in the background through Android's GoogleFit service upon approval from the participant (optional along ICF)
		Calories burned	Analysis of calories burned through activity
		Move Minutes	Number of minutes the user has moved as collected through smartphone and/or connected devices and calculated by GoogleFit
		Step count cadence	Measures steps per minute. Each data point represents an instantaneous measurement of the cadence in steps per minute.

		Step count delta	Captures the number of steps taken since the last reading.
		Speed	Walking speed calculated based on steps per minute.
**3. Sensor data from smartphone**			
	iOS and Android devices		Data is captured from the device ONLY when the participant is performing the DREAMS challenges
		Accelerometer	An accelerometer measures changes in velocity along one axis.
		Gyroscope	A gyroscope measures the rate at which a device rotates around a spatial axis. Devices selected have a three-axis gyroscope, which delivers rotation values in each of the three axes
		Magnetometer	Provides compass-level orientation data and raw magnetometer data. It provides measurements of the Earth's magnetic field relative to the device.
		Altimeter	Provides altitude data based on barometric sensor information.
		Pedometer	Provides step-counting data from the built-in motion processor.
**4. Fitbit (processed data, no raw sensor data)**			
	Activity analysis:		Results from activity analyses based on Fitbit sensors.
		Calories	Calories based on tracked activity
		Calories BMR	Calories based on tracked activity, but only basal metabolic rate calories
		Activity Calories	Calories based on tracked activity, but only based on data from tracker (not including manual data)
		Steps	Number of steps
		Distance	Distance covered
		Floors	Calculation of elevations in terms of floors (based on altimeter data)
		Elevation	Elevation as measured by altimeter

		Minutes Sedentary	Number of minutes of activity in a sitting positions
		Minutes of light, fair and intense activity	
		Heart Rate	Inter- and intra-day heart rate
		Nutrition	Nutrition information, if provided by participant
		Weight	Weight, if and when entered by participant or connected scale
		Profile	Profile data (may contain personally identifiable information (PII), if provided by participant
	Sleep analysis*:		Results from sleep analyses based on Fitbit sensors
		Date	
		Duration	
		Efficiency	Total sleep time/total time in bed
		Stages	Sleep Stages as defined by Fitbit: deep, light, rem and wake
		Minutes in bed after wake up	
		Minutes registered as awake in bed	
		Minutes from getting in bed to falling asleep	
		Start time in bed	
		Total time in bed, asleep and awake	

Table 4: Data points that are collected through the DREAMS App

*Participants will be asked to wear the Smartwatch while sleeping but not to take the Smartphones to bed.

3.2.7 Precautions for use

DREAMS is intended for patients with MS and sex- and age matched healthy controls as defined with the in- and exclusion criteria.

3.2.8 Software Safety Classification

- Class A, hazardous situation will not arise from failure of the software.
- There is no contact with body and/or fluids.

3.2.9 User training

Study participants will be instructed and trained by a qualified Study Nurse at the Baseline Visit (V2) of this Feasibility Study.

3.3 Preclinical Evidence

No non-clinical data is available.
The DREAMS App is a low risk (class I) device. The smartphone App has no direct body contact and does not have biological effects as described in ISO 10993.

3.4 Clinical Evidence to Date

A number of clinical trials have been performed, or are in progress, with similar devices, e.g.:

- MSCopilot (*NCT03148938*)
- Floodlight, including Floodlight Open (e.g. https://floodlightopen.com/de-CH/)
- MyMS (NCT03111394)

The results from the completed studies do not indicate incidents or adverse effects on patients. No post marketing experience is available yet.

3.5 Medical Device: Rationale for the intended purpose in study (pre-market MD)

3.5.1 Introduction

The study team aims to capture 10 data repeats per task and study participant for all tasks within the 6 weeks study period. A weekly schedule has been developed and supports that goal (see table 5). The schedule is developed such that participants are requested to perform different challenges every day to prevent a high level of repetition.

- Challenges must be performed during 5 days every week, participant can select which 5 days work best for him/her
- Total time spent on performing scheduled exercises should be maximum 15-20 minutes per (active) day
- The schedule should minimize having multiple strenuous exercises ("La promenade", "Musical Chairs" and "Spanische Treppe") on one day. If the schedule is too demanding for one day, participants can defer challenges to another day.
- Study nurse will be alerted if user completes < 75% of exercises (i.e. missing >2) for at least 2 out of 5 days
- Users can choose the order of challenges for one day, challenges are categorized in activity challenges and cognitive games
- Only fully completed challenges are registered, marked as completed and sensor data is collected successfully. For not completed challenges information about the event is recorded for further improvement of the app development and sensor data is not collected.
- Users will not be able to perform challenges or play games outside the schedule
- Study nurse will train the participant to make sure that all tasks are fully understood and are performed correctly
- I can't do this:
    - User can indicate that he/she wants to postpone exercise to later in the week (one of the 2 free days)
    - Maximum of 4 exercises can be moved to each of these 2 days
- System of reminders is in place for participants to complete daily schedule

Table 5

Challenge schedule: Weeks 1 - 6								
			Weeks 1-5					Week 6
#	Challenge Name	Time (sec)	Day 1	Day 2	Day 3	Day 4	Day 5	
1	La Promenade	120	120			120		
2	Musical chairs	30			30		30	
3	The Butler	40	40				40	
4	Pinguin	20			20		20	
5	Spanische Treppe	40		40		40		
6	Schweizergarde	60			60		60	
7	Catch-A-Cloud	30			30		30	
8	Konfetti	60	60			60		
9	PEAK - Flame Dodge left hand	60			60		60	
10	PEAK - Flame Dodge right hand	60			60		60	
11	mSDMT (voice)	90	90					
12	PEAK - Word Hunt	180		180			180	
13	PEAK - Spin Cycle	60		60			60	
14	PEAK - Zap Gap	60		60		60		
15	PEAK - Face Switch	60	60				60	
16	PEAK - Rush Back	60			60		60	
17	PEAK - Baggage Claim	180		180		180		
18	PEAK - Perilous Path	180	180		180			
19	PEAK - Puzzle Blox	60	60		60			
20	PEAK - Must Sort	60		60		60		
21	PEAK - Low Pop	60		60		60		
22	Adlerauge	30			30			
23	Nebel	30			30			
24	MSIS-29	120						Day 1
25	MSWS-12	180						Day 2
26	Fatigue Severity Scale	120						Day 2
27	MS Symptom Tracker	120						Day 3
28	Feedback Questionnaire and Semi-Structured Interview	1'800						Day 4 or 5
	Total time per day (min)		10.2	10.7	10.3	9.7	11.0	Day 1: 10<br>Day 2: 10<br>Day 3: 10

								Day 4 or 5: 40
	Number of challenges		7	7	11	7	11	

Table 5: Weekly Schedule

3.6 Explanation for choice of comparator (or placebo)

Not applicable.

3.7 Risks / Benefits

3.7.1 Risk analysis

A risk assessment has been performed according to EN ISO 14971 (see Appendix Risk Management 17.6.). The results are presented below.
The risk assessment shows that no unacceptable residual risks are identified. Mitigating controls and procedures have been identified to any risk higher than acceptable risks.
Concerning Data Safety, Data Flow and Analysis an internal (University Hospital Basel (USB)) consortium was established and alignment with data security officers (university and hospital) as well as the legal head of the hospital was achieved.

Summary

The risk analysis concludes that no unacceptable residual risks are identified and no serious adverse effects are expected from the use of the device or participation in the study.
The study is not expected to directly benefit the study participants. But the results will enable the sponsor to perform the following validation study and therefore contribute to the development of reliable and valid digital biomarkers as a promising tool to improve patient monitoring and management.

3.7.2 Post-trial care

No post-trial care is needed, as this is not an interventional study.

3.7.3 Competing studies

No validated and comprehensive suite of digital markers exists up to now. As mentioned in section 3.1 the DREAMS App is unique by its comprehensive set of active challenges, gamified neuropsychological assessment, passive monitoring and questionnaires. The systematic approach with a clinical feasibility study and the following validation study will ensure, that the selection of biomarkers will enable better monitoring and eventually better and personalized treatment of PwMS.

3.8 Justification of choice of study population

Patients under the care of our Multiple Sclerosis Center who are representative for the general MS population will be approached and included (age range 18-70, EDSS 0-6.5) In addition a group of age- and sex- matched healthy controls will be included.
Patients and Healthy controls will need to possess a Smartphone and be able to use the App (sufficient vision, no major cognitive deficits, no major motor and dexterity impairment that would preclude safe administration of the App challenges).

4. STUDY OBJECTIVES

4.1 Overall Objective

The objective of this feasibility study is to identify digital biomarkers that are technically reliable and measurable as well as meaningful to patients with MS.

4.2 Primary Objective

The primary objective of this study is to identify digital biomarkers that are technically reliable and measurable, and perceived as user friendly and meaningful for patients with MS (PwMS).

4.3 Exploratory Objectives

The exploratory objective of this study is to find differences on these biomarkers between 30 PwMS and 30 age- and sex- matched healthy controls (HC) on a group level.

4.4 Safety Objectives

Not applicable.

5. STUDY OUTCOMES

5.1 Primary Outcome

<u>The primary study outcomes are:</u>
1. Test reliability of digital biomarkers measured by Intra Class Correlation (ICC≥0.6)
2. Test reliability of digital biomarkers measured by Coefficient of variation (CV< 20%)
3. Determination of user acceptance of digital biomarkers with regards to acceptance based on questionnaire (>3 on a Likert Scale)

5.2 Exploratory Outcomes

<u>Exploratory study outcome is to identify digital biomarkers that:</u>
a.) differenciate on a group level between PwMS and age- and sex- matched healthy controls (HC).
b.) correlate with already established assessment tools in their respective domains (e.g. "Catch-a-cloud" vs. Nine-Hole-Peg test).

5.3 Other Outcomes of Interest

-

5.4 Safety Outcomes

Not applicable.

6. STUDY DESIGN

6.1 General study design and justification of design

This feasibility study is a prospective observational proof of concept study in patients with MS with a control group of healthy volunteers. Duration of the study will be 6 weeks per patient. This study is the first step to identify precise, well accepted and meaningful digital biomarkers for patients with multiple sclerosis as a first step towards validation in further studies. Digital biomarkers will be captured by patient's own smartphones (iOS and Android) and by an additional smartwatch (Fitbit Versa 2). The smartwatches will be given to all participants and will be handed over to them after the study end (market value CHF 210/smartwatch).
30 patients with MS and 30 age- and sex- matched healthy controls will be included. All participants will be instructed by a study nurse at the beginning of the study to assure that all patients and healthy controls will use the App and perform all tasks in a similar and correct way. Within the study all participants will perform all tests repeatedly over a time period of 5 weeks (every test twice a week resulting in 10 repetitive performances/test). In week number 6 they will perform all questionnaires and will have their semi-structured interview.

This feasibility study aims to select the most reliable and meaningful biomarkers out of over 20 candidate biomarkers developed by our team.
Ranking of test performance will be done for each of the digital biomarkers by:
- Intra Class Correlation of at least 0.6 if repeated 10 times within 6 weeks
- Coefficient of variation must be less than 20% if repeated 10 times within 6 weeks
- User acceptance as assessed by a feedback questionnaire and a semi-structured interview at the end of the study (>3 on a likert scale).

For detailed criteria of performance see Chapter 11. Statistical Methods, 11.4.2. Primary Analysis.

For the exploratory analysis (potential differences between healthy controls and PwMS on a group level and correlation with already established assessment tools in their respective domains) age- and sex matched healthy controls will be included who will follow an identical test schedule and also provide their feedback about the App.

6.2 Methods of minimising bias

Not applicable.

6.2.1 Randomisation

Not applicable.

6.2.2 Blinding procedures

Not applicable.

6.2.3 Other methods of minimising bias

Not applicable.

6.3 Unblinding Procedures (Code break)

Not applicable.

7. STUDY POPULATION

7.1 Eligibility criteria

Participants fulfilling all of the following <u>inclusion</u> criteria are eligible for the study:

A. Participants with MS
- Age 18-70
- Diagnosed with MS according to the revised McDonald criteria 2017[1], all clinical forms inclusive (CIS, RRMS, SPMS, PPMS)
- EDSS ≤ 6.5
- In possession of a DREAMS App compatible smartphone (iOS/Android, see section 3.2.4, Table 2)
- Corrected close visual acuity of ≥0.5
- Hand motor skills sufficient for using a smartphone
- Ability to follow the study procedures
- Informed Consent as documented by signature

B. Healthy controls (sex and age matched to patients)
- Age 18-70
- In possession of a DREAMS App compatible smartphone (iOS/Android, see section 3.2.4, Table 2)
- Corrected close visual acuity of ≥0.5
- Hand motor skills sufficient for using a smartphone
- Being able to walk without aid
- Ability to follow the study procedures
- Informed Consent as documented by signature

The presence of any one of the following <u>exclusion</u> criteria will lead to exclusion of the participant:

A. Participants with MS
- Other clinically significant concomitant disease states (e.g., renal failure, severe hepatic dysfunction, severe/unstable cardiovascular disease, progressive cancer, etc.)
- Known or suspected non-compliance, drug or alcohol abuse
- Women who are pregnant or breast feeding
- Being a frequent PEAK* user (having used PEAK ≥ once daily over a period of ≥3 weeks) and not willing not to use PEAK during the study period

* PEAK: freely available App for cognitive training

B. Healthy controls (sex and age matched to patients)
- Being diagnosed with MS or other disease affecting neurological and cognitive functions
- Other clinically significant concomitant disease states (e.g., renal failure, severe hepatic dysfunction, severe/unstable cardiovascular disease, progressive cancer, etc.)

- Known or suspected non-compliance, drug or alcohol abuse
- Women who are pregnant or breast feeding
- Being a frequent PEAK* user (having used PEAK ≥ once daily over a period of ≥3 weeks) and not willing not to use PEAK during the study period

* PEAK: freely available App for cognitive training

7.2 Recruitment and screening

Patients will be recruited at the MS Center Basel (Department of Neurology, University Hospital Basel, Basel, Switzerland).
Patients will be asked during their (regular) clinical visits to participate, will be provided with information and will be asked to think about a possible participation. Once willing to participate, they will be invited for the screening visit. The clinical information will be documented from the latest clinical visit (patient history, medication, EDSS, MSFC (Multiple Sclerosis Functional Composite (T25FW, 9-HPT)) and SDMT (Symbol Digit Modalities Test)).
If patients are included and some of the tests have not already been performed, these will be performed at Screening or Baseline. In addition to the standard tests we will also perform a neuropsychological assessment (see chapter 9 study assessment), near vision acuity and contrast vision assessment.
Healthy controls will be recruited via advertisements and amongst relatives of patients attending the clinic and will be invited for screening if willing to participate. Healthy controls have to be included sex- and age- matched to patients and therefore will be recruited accordingly.

7.3 Assignment to study groups

Not applicable.

7.4 Criteria for withdrawal / discontinuation of participants

Patients and healthy controls will be withdrawn from the study if the following occurs during the study period:

- Other clinically significant concomitant disease states (e.g., renal failure, severe hepatic dysfunction, severe/unstable cardiovascular disease, progressive cancer, etc.)
- Non-compliance
- Inability to follow the procedures of the study, e.g. due to language problems, psychological disorders, dementia, etc. of the participant
- Pregnancy: If a participant will realise pregnancy during the study period, she should contact the study nurse. The participant will be allowed to continue the study if she wants to but of course can also withdraw consent immediately.
- Loss of their DREAMS App compatible smartphone (iOS/Android, see section 3.2.4, Table 2)
- Close visual acuity loss of < 0.5
- Loss of hand motor skills not sufficient for using a smartphone
- Using PEAK* during the study period

* PEAK: freely available App for cognitive training

Withdrawn participants (drop-out) will be replaced by an additional participant.
Data of withdrawn participants collected until withdrawal will not be deleted and will be used in a coded form for research purposes.

8. STUDY INTERVENTION

8.1 Identity of Investigational Products (treatment / medical device)

8.1.1 Experimental Intervention (treatment / medical device)

DREAMS is a mobile application for PwMS and a central data processing unit in a secure computer environment, and a platform that supports study administration.
DREAMS R1.4 is designed and developed to support the feasibility study. During the feasibility study, the study team will evaluate a wide range of digital BioMarker (dBM) candidates as well as technical stability of the device. During the prospective, non-randomized validation study to follow the feasibility study, the study team will evaluate a selection of the most promising digital BioMarker candidates during a cross-sectional and longitudinal study.
Graph 3:

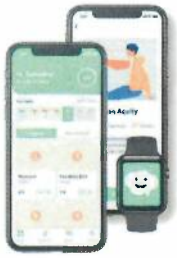

Graph 3: DREAMS App

During the study, users are requested to perform a series of tasks ("challenges"). During the challenges, the smartphone's sensors will gather data that can be analysed to measure the participants' performance and provide an indication of the level of impairment. Table 6 below summarises each of the challenges and the objectives for these challenges. The cognitive games are provided by PEAK (peak.net) a cognitive game App developer.

Table 6

#	Challenge Name	Short description	Objective(s)
1	La Promenade	Walk briskly for 2-minutes, without break	Measure speed, gait and balance
2	Musical chairs	Stand-up and sit-down on a chair for 30 seconds	Measure number of completed movements and balance
3	The Butler	Keep arm up for 10 seconds each, first with eyes open and then with eyes closed	Measure how well user can keep arms up as well as postural tremor in upper limbs

#	Challenge Name	Short description	Objective(s)
4	Pinguin	Stand with arms along the body for 10 seconds, first with eyes open, then with eyes closed	Measure balance impairment
5	Spanische Treppe	Walk up and down a set of stairs, answer a few questions regarding the exercise	Measure functional strength, balance and agility
6	Schweizergarde	Make a U-turn after each 5 steps taken	Measure the time it takes to make 5 turns, as well as gait and balance
7	Catch-A-Cloud	Touch the moving cloud with index finger as often as possible.	Measure the number of touches and the level of accuracy for each attempt
8	Konfetti	Keep phone in arm and bend such that the tip of the nose touches the bulls eye on the screen, with both arms, eyes open and closed	Measure how frequent and accurate the user touches the screen, as well as intention tremor
9	PEAK - Flame Dodge left hand	Move a character through a series of numbers, while avoiding flames – with the left hand	Measure hand-eye coordination
10	PEAK - Flame Dodge right hand	Move a character through a series of numbers, while avoiding flames – with the right hand	Measure hand-eye coordination
11	mSDMT (oral)	Call out the correct number for the presented symbols using a key.	Measure processing-speed of information
12	PEAK - Word Hunt	Find words in a diagram	Measure language skills
13	PEAK - Spin Cycle	Observe and recognise changing patterns	Measure working-memory
14	PEAK - Zap Gap	Sort falling orbs by colour	Measure mental agility
15	PEAK - Face Switch	Correctly answer questions about presented faces	Measure mental agility and Inhibition
16	PEAK - Rush Back	Memorize and recall symbols on cards	Measure focus
17	PEAK - Baggage Claim	Memorize cities and recall them after an distractor challenge is presented	Measure memory, with distraction
18	PEAK - Perilous Path	Memorise the mines to find a connection between dots without touching the mines	Measure visuo-spatial memory
19	PEAK - Puzzle Blox	Match a presented shape from a selection of blocks	Measure problem solving

#	Challenge Name	Short description	Objective(s)
20	PEAK - Must Sort	Sort items by clicking left of right	Measure focus
21	PEAK - Low Pop	Tap tiles with random numbers from lowest to highest	Measure prioritizing and speed
22	Adlerauge	Acuity vision test, for both eyes	Measure (near) acuity vision (40 cm)
23	Nebel	Contrast vision test, for both eyes	Measure contrast vision (40 cm)
24	MSIS-29	Multiple Sclerosis Impact Scale, see Appendix 17.5.1.1.	Validated questionnaire (DE version)
25	MSWS-12	Multiple Sclerosis Walking Scale, see Appendix 17.5.1.2.	Validated questionnaire (DE version)
26	Fatigue Severity Scale	Fatigue Severity Scale, see Appendix 17.5.1.3.	Validated questionnaire (DE version)
27	MS Symptom Tracker	Questionnaire regarding symptoms PwMS has experienced in the last 2 weeks, see Appendix 17.5.1.4.	Identification of experienced symptoms. Developed by study team.
28	Feedback Questionnaire	Questionnaire with regards to the acceptance and relevance of the challenges presented in the mobile app	User feedback. Developed by study team.

Table 6: Description of Challenges

8.1.2 Control Intervention (standard/routine/comparator treatment / medical device)

There is no control intervention.

8.1.3 Packaging, Labelling and Supply (re-supply)

DREAMS is a Software as a candidate Medical Device (SaMD) solution. The solution is provided to the study participant via a mobile application (App) on the participant's smartphone, which will be made available via the app stores for Apple (App Store) and Android (Google Play). The study nurse will support the study participant in downloading and setting up the required user account and schedule. Once the App is set up, no further maintenance is required by the study participant.
The App is designed for intended use by lay persons and has examples and instructions that guide the users through each of the functions and challenges. In case the study participant has questions regarding the use of the App or any of the activities, the study

nurse is available for first line support and the manufacturer's support team is available for technical support.
Information about the App, manufacturer and version are included in the "about DREAMS" function in the App. This version of the App is developed for this study, the App is not available to the wider public yet and is not yet registered as a Medical Device.

8.1.4 Storage Conditions

Not applicable.

8.2 Administration of experimental and control interventions

8.2.1 Experimental Intervention

Every participant will upload the same version of the DREAMS App (see sections 3.2.5. and 8.1.1) and will perform the same test schedule (see section 3.5.1. Table 5). Every participant will be instructed by the study nurse (App guidance and challenge instructions).

8.2.2 Control Intervention

There is no control intervention planned.

8.3 Dose / Device modifications

Not applicable

8.4 Compliance with study intervention

Study nurse will be alerted if user completes < 75% of exercises (i.e. missing >2) for at least 2 out of 5 days. The study nurse will contact participants by phone if this is the case to offer help. Participants can contact the study nurse and the study physician anytime. Non-compliance (sustained non-adherence to completion of the scheduled exercises) will result in withdrawal. After withdrawal we will try to get withdrawn participants feedback (semi-structured interview) for understanding the reason for non-compliance.

8.5 Technical Support

There will be a technical support from our main development partner Healios GmbH for the clinical team (study nurse, study physician and all investigators). IT issues will be classified the same day into two main categories: "critical" or "non critical". "Critical" IT issues will be addressed the same day, "non critical" IT issues within two business days. The Healios team is not permitted to contact the participants directly.

8.6 Data Collection and Follow-up for withdrawn participants

Data from withdrawn participants (drop out) will be included into analysis (intention to treat analysis). Withdrawn participants will be asked if they would be willing to be invited for the feedback interview in order to learn from their experience with the App. This will take place in study visit V3. The study design has no official follow-up visit.

8.7 Trial specific preventive measures

Not applicable.

8.8 Concomitant Interventions (treatments)

Not applicable.

8.9 Study Drug / Medical Device Accountability

DREAMS is a Software as a Medical Device solution. 'Shipment' of the mobile application is performed via the Apple and Android App store. The study team controls that only participants that have signed an Informed Consent Form can access the App. Access is further controlled through use of a personal user-ID, that is provided by the study team, and a unique password.

Admission of the activities is scheduled and managed through the central database and processing unit. The study nurse that is available to the study nurse has functionality to monitor if the study participant complies with the weekly schedule.

8.10 Return or Destruction of Study Drug / Medical Device

Upon completion of the study the App will no longer be accessible for the study participant. Blocking access is managed centrally through the central processing unit.

9. STUDY ASSESSMENTS

9.1 Study flow chart(s) / table of study procedures and assessments

Table 7

		Week -1	Week 1	Week 2	Week 3	Week 4	Week 5	Week 6	
**Visit Name**	**S**	**BL**							**End of Study**
**Visit Number**	**V1**	**V2**							**V3**
Participant **Information and Informed Consent**	+								
**In- /Exclusion Criteria**	+								
**Medical* History**	+								
**App download and test instructions**		+							
**Vision tests (contrast vision inclusive)**		+							
**EDSS* (PwMS only)**		+							+
**MSFC, SDMT***		+							
**Neuro-psychological Testing**		+							
**Test performed by study participants (App active and passive monitoring)**			+	+	+	+	+		
**Questionnaires**								+	
**Feedback Questionnaire and Semi-Structured Interview**									+

Table 7: Overall Study Assessment Schedule

S=Screening, BL=Baseline, * Data will be taken of routine visit if this visit is +/- 2 weeks around BL. If this is not feasible data will be collected the latest at BL. EDSS = Expanded Disability Status Scale, MSFC= Multiple Sclerosis Functional Composite (T25FW, 9-HPT), SDMT= Symbol Digit Modalities Test.

A. Non App Related

Once patients and healthy controls have been included into this study the following information will be collected and tests performed (not App related):

Table 8

	Patients with MS	Healthy Controls
1. Demographic Data	+	+
2. Medical History (medication inclusive)	+	+
3. EDSS	+	
4. MSFC	+	+
5. SDMT	+	+
6. Near visual acuity	+	+
7. Contrast vision	+	+
8. Neuropsychological Testing	+	+

Table 8: Information collected

EDSS = Expanded Disability Status Scale, MSFC = Multiple Sclerosis Functional Composite (T25-FW, 9-HPT), SDMT = Symbol Digit Modalities Test.

1. Demographic Data

Sex, DOB (Date of Birth), Age, height & weight (BMI), race, education, employment, dextrality (right/left)

Education & Employment (detailed list see CRF):

Table 9

Education (years in school)	
Employment	

Table 9: Questions about Education & Employment, details see CRF

2. Medical History

List of current diagnosis, list of current medication (name, dosing, frequency, since when) and past medication (last two years), family history of diseases (especially MS, ADEM, NMO, ON, Myelitis, other demyelinating diseases)

For Patients with MS: Type of MS (CIS, RRMS, SPMS, PPMS), Date of first symptoms, date of diagnosis, year of transition to RRMS or/and SPMS (if applicable), Attack history (last two years), DMT & symptomatic treatment.

3. EDSS

Expanded Disability Status Scale. For patients with MS only, see https://www.neurostatus.net/ . EDSS will be performed at BL (V2) and at end-of-study visit (V3).

4. MSFC

Multiple Sclerosis Functional Composite (T25FW, 9-HPT)

5. SDMT
SDMT = Symbol Digit Modalities Test.

6. Near Vision Acuity
Will be assessed with a common near vision chart:
https://www.ophtha-shop.ch/de/A~09.20.10E/Nahsehprobentafel-Mod.-2306-E-Haken-40cm-mit-Distanzkordel

7. Contrast Vision
Will be assessed using a Pelli-Robson Trans-Illuminated Contrast Sensitivity Chart for Low Vision and Peak CS with Landolt C optotypes for an ETDRS Illuminator Cabinet (Model 2425E): https://www.precision-vision.com/products/contrast-sensitivity-tests/peak-contrast-sensitivity/pelli-robson/pelli-robson-trans-illuminated-contrast-sensitivity-chart/?variant=19834

8. Neuropsychological Testing
The neuropsychological test-battery will consist of:

Table 10

Test Name	Test Explanation
ROCF (Rey-Osterrieth Complex Figure Test)	The ROCF tests visuo-constructive abilities, visual memory and executive functioning (mainly planning). Participants are asked to copy and memorize a complex figure, then draw from memory after ~ 3 minutes and draw once more after ~ 30 minutes.
VLMT (Verbaler Lern- und Merkfähigkeitstest) german version of the «Rey auditory verbal learning test»	The VLMT tests verbal learning and memory. The test-rater reads 15 unrelated words, which the participants must memorize and recall immediately. After 5 repetitions (always the same words) an interference list is read once (15 new words, which the participant must memorize and recall as well). Immediately thereafter the participant must recall the first wordlist from memory and again after 25-30 minutes.
MUSIC (Multiple Sclerosis Inventory of Cognition)	The MUSIC functions as a screening tool for general cognition in MS patients. The screening consists of 5 subtests assessing verbal memory, mental flexibility/set-shifting, information processing speed and inhibition. Additionally, there is a short fatigue screening (3-item questionnaire) included.
STROOP Test	The STROOP test measures processing speed and inhibition ability. The participant is first presented with 24 words written in black ink spelling colours, they have to read the words as fast as they can. In a second round they only see coloured dots/lines and have to name the colour of each dot/line as fast as they can. In the third round, participants are presented with 24 coloured words spelling colours (colour of the word ≠ spelled colour of the word). The task is to name the colours the words are printed in as fast as possible.
TMT (Trail Making Test A&B)	The TMT tests aspects of executive functioning such as attention, planning and mental flexibility. In part A the participants are asked to connect numbers (randomly spread out on a sheet) with a pencil as fast as they can. In part B there is an additional

	sequence (alphabet), also randomly spread out on the sheet. The participants must now connect switching from number to letter (e.g. 1-A-2-B-3-C...) as fast as they can.
HADS (Hospital Anxiety and Depression Scale)	The HADS is a 14-item questionnaire screening for anxiety and depression symptoms. Both subgroups anxiety and depression have 7 items that can be rated from 0-3 (0= normal, 3= significant symptom), with a max. of 21 points per subscale. A higher score associates with a worse symptomatology. A score up to 7 is regarded as normal, 8-10 points is interpreted as potential signs of anxiety/depression and a score of over 10 indicates definite clinically significant symptoms of the disorder. With a score of over 10 it is recommended to seek help and further investigate the probable existence of a disorder.
EMS (Explorationsmodul MS)	The EMS is a questionnaire for self-reported quality of life. It is split into 4 subgroups: emotion, cognition, fatigue and psycho-social factors. There are 3 questions per subgroup, which can be answered either "yes" (1 point) or "no" (0 points). The EMS is rather used as an overview of the current wellbeing of the pwMS than as a diagnostic tool. It also helps monitor the disease progression.

Table 10: Neuropsychological Testing

B. App related

At Screening (V1) Informed Consent will be sought for every participant once it is decided to include them into the study. At BL (V2) the App will be downloaded by the study nurse and patients will receive a Code Number. There will be a detailed instruction from the study nurse concerning the App and all tests to be performed.
During Feasibility Study:

- Exercises must be performed during 5 days every week, participant can select which 5 days work best for him/her
- Total time spend on performing scheduled exercises should be maximum 15-20 minutes per (active) day
- The schedule should minimize having multiple strenuous exercises ("La promenade", "Musical Chairs" and "Spanische Treppe") on one day. If the schedule is too demanding for one day, participants can defer challenges to another day.
- Study nurse will be alerted if user completes < 75% of exercises (i.e. missing >2) for at least 2 out of 5 days
- Users can choose the order of activities for one day, activities are categorized in activities and cognitive games
- Only fully completed challenges are registered, marked as completed and sensor data is collected successfully. For not completed challenges information about the event is recorded for further improvement of the app development and sensor data is not collected.
- Users will not be able to perform exercises or play games outside the schedule
- Study nurse will train the participant to make sure that all tasks are fully understood and are performed correctly

- I can't do this:
  - User can indicate that he/she wants to postpone exercise to later in the week (one of the 2 free days)
  - Maximum of 4 exercises can be moved to each of these 2 days
- System of reminders is in place for participants to complete daily schedule

9.2 Assessments of outcomes

See sections 5 Study Outcomes, 8.2.1. Experimental Interventions and 11. Statistical Methods.

9.2.1 Assessment of primary outcome

Please see sections 5.1., 8.2.1., 11 and 12.2.

9.2.2 Assessment of exploratory outcomes

Please see sections 5.2., 8.2.1., 11 and 12.2.

9.2.3 Assessment of other outcomes of interest

Please see sections 5.3., 8.2.1., 11 and 12.2.

9.2.4 Assessment of safety outcomes

At the End-of-Study visit (V3) participants will be asked if they experienced any kind of safety issues.

9.2.4.1 Adverse events

At the End-of-Study visit (V3) participants will be asked if they experienced any kind of Adverse event. The event will be described in detail and the potential relationship to the medical device/clinical study procedure will have to be defined (certain, probable, possible, unlikely).

9.2.4.2 Laboratory parameters

Not applicable.

9.2.4.3 Vital signs

Not applicable.

9.2.5 Assessments in participants who prematurely stop the study

Data from withdrawn participants (drop out) will be included into analysis (intention to treat analysis). Withdrawn participants will be asked if they would be willing to be invited

for the feedback interview in order to learn from their experience with the App. The study design has no official follow-up visit.

9.3 Procedures at each visit

See Chapters 8.2.1. Experimental intervention and 9.1. Study Flow Chart.

9.3.1 Split into subtitles by type of visit

For details see also section 9.1.

Screening Visit (V1):

- Informed Consent
- Study portal registration
- Randomized subject ID will be created
- Screening examinations (vision test, dexterity)
- Check In- and Exclusion Criteria
- Medical History Part 1
- Final decision of Inclusion into the study

Baseline Visit (V2):

- Demographic Data
- Medical History Part 2
- EDSS (if not already performed)
- MSFC and SDMT (if not already performed)
- Neuropsychological Testing
- Contrast Vision Test
- DREAMS App download and test instructions

End of Study Visits (V3)

- EDSS
- Feedback questionnaire and semi-structured interview.
- The occurrence of any Adverse Event during the study period will be explored.
- The Fitbit Versa 2 Smartwatch will be handed over to the participants.
- The study conclusion will be drawn (study completed by participant according to the protocol)

10. SAFETY

10.1 Medical Device Category C studies

Device deficiencies and all adverse events (AE) including all serious adverse events (SAE) are collected, fully investigated and documented in the source document and appropriate case report form (CRF) during the entire study period, i.e. from patient's informed consent until the last protocol-specific procedure, including a safety follow-up period [ISO 14155]. Documentation includes dates of event, treatment, resolution, assessment of seriousness and causal relationship to device and/or study procedure.

10.1.1 Foreseeable adverse events and anticipated adverse device effects

Potential adverse events:

Table 11

**Risk (R)**	**Function of Device**	**Hazard (root cause)**	**Potential causes of failure**	**Result of failure or use**	**Harm**
**R1***	Motor skills	Participant loses balance	(worsening) handicap of participant, by accident	Participant falling	Bruise, broken limb
R2	IT systems	App failure to function	-no wifi connectin<br>-back-end not available	Participant not able to perform acitvities	Nuisance and impact on study execution
R3	Device	Sensors not reporting accurately	Device failure	- participant may have to redo activity<br>- recorded data inaccurate	Nuisance and impact on study execution
R4	Device	App failure to function	Smarphone failure	- no data	Nuisance and impact on study execution
R5	Smartwatch	Skin irritation	Skin sensitivity	Skin irritation	Skin irritation
**R6****	Security	Data privacy breach	- Hacker has access to Healios systems<br>- Third party has access to patients phone	Data exposed to unautho-rised person	Privacy breach
R7	Instructions	Instructions are not clear	Instructions are not clear	Tests are not executed as intended	Nuisance and impact

					on study execution
R8	Labelling issues	n/a	n/a	n/a	n/a
R9	IT systems	App malfunctions	Changes or errors in third party and/or open libraries used	-partici-pant is not able to perform activities<br>- Outcomes may not be presented accurately	Nuisance and impact on study execution
R10	IT Systems	Interference with other devices/Apps	Interference with other devices/Apps	Interference with other devices/Apps	Bad user experience
R11	General	Biocompatibility issues	n/a	n/a	n/a

Table 11: Potential adverse events DREAMS App. n/a= not applicable

***R1 will be prevented by a very careful instruction at BL. If any safety concern exists participant will not be included or will be included but challenge with safety concerns will not have to be performed (can be deleted individually on the schedule by the study nurse).**
****R6 will be prevented by very careful established security guidelines.**

10.1.2 Definition and Assessment of (Serious) Adverse Events and other safety related events

Definitions:

- *Adverse Event* (AE)

Any untoward medical occurrence, unintended disease or injury or any untoward clinical signs (including an abnormal laboratory finding) in participants, users or other persons whether or not related to the investigational medical device [ISO 14155: 3.2].
NOTE 1 This definition includes events related to the investigational medical device or the comparator.
NOTE 2 This definition includes events related to the procedures involved.

NOTE 3 For users or other persons, this definition is restricted to events related to investigational medical devices.

- *Adverse Device Effect* (ADE)

Adverse event related to the use of an investigational medical device [ISO 14155: 3.1].
NOTE 1 This definition includes adverse events resulting from insufficient or inadequate instructions for use, deployment, implantation, installation, or operation, or any malfunction of the investigational medical device.
NOTE 2 This definition includes any event resulting from use error or from intentional misuse of the investigational medical device.

- *Serious Adverse Event* (SAE) [ISO 14155: 3.37]:

Any adverse event that led to any of the following:

(a) death,

(b) serious deterioration in the health of the subject that resulted in any of the following:

(i) life-threatening illness or injury,

(ii) permanent impairment of a body structure or a body function,

(iii) hospitalisation or prolongation of patient hospitalisation,

(iv) medical or surgical intervention to prevent life-threatening illness or injury or permanent impairment to a body structure or a body function,

(c) foetal distress, foetal death or a congenital physical or mental impairment or birth defect.

NOTE 1 Planned hospitalization for a pre-existing condition, or a procedure required by the CIP, without serious deterioration in health, is not considered a serious adverse event.

- *Device deficiency*:

Inadequacy of a medical device related to its identity, quality, durability, reliability, safety or performance, such as malfunction, misuse or use error and inadequate labelling [ISO 14155: 3.15].

- *Device deficiency with SAE potential*:

Device deficiencies that might have led to a serious adverse event if a) suitable action had not been taken or b) intervention had not been made or c) if circumstances had been less fortunate.

- *Health hazards that require measures*:

Findings in the trial that may affect the safety of study participants and, which require preventive or corrective measures intended to protect the health and safety of study participants SAE [ClinO Art. 37].

- *Causal Relationship of SAE* [MEDDEV 2.7/3 revision 3, May 2015]:

A causal relationship towards the medical device or study procedure should be rated as follows:

- **Not related:** The relationship to the device or procedures can be excluded.
- **Unlikely:** The relationship with the use of the device seems not relevant and/or the event can be reasonably explained by another cause, but additional information may be obtained.
- **Possible:** The relationship with the use of the investigational device is weak but cannot be ruled out completely. Alternative causes are also possible.
- **Probable:** The relationship with the use of the investigational device seems relevant and/or the event cannot reasonably explained by another cause.
- **Causal relationship:** The serious event is associated with the investigational device or with procedures beyond reasonable doubt.

Device deficiencies that might have led to an SAE are always related to the medical device.

Assessments:

Any AE, ADE, SAE or Device Deficiency will be analysed and reported carefully during the whole study period. Measures will be taken accordingly (see 10.1.3). At end of study visit (V3) every participant will be interviewed by the study nurse if any additional AE, ADE, SAE or Device Deficiency has occurred not already mentioned up to V3.
Participants are informed that they should report any event immediately to the study nurse by calling or mailing the study nurse. The study nurse will record the report in the CRF and determine if it is a (Serious) Adverse Event, ADE or Device Deficiency.
The study nurse, or other study team member, will report to the PI within 24 hours of the event:

- All SAEs
- Device deficiencies

The Sponsor-Investigator will evaluate SAEs with regard to causality and seriousness. Device deficiencies are assessed regarding their potential to lead to an SAE.
Device deficiencies will also be reported to the Manufacturer (Healios).
In addition, the DREAMS App has a relapse reporting process, see Appendix 17.5.1.5. All relapses reported are captured in the Nurse Portal and will be followed up by the study nurse.
The study team will also ask all study participant for any (Serious) Adverse Events during the End of Study visit (Visit 3).

10.1.3 Reporting of (Serious) Adverse Events and other safety related events

Reporting to Sponsor-Investigator:

The following events are to be reported to the Sponsor-Investigator within 24 hours upon becoming aware of the event:

- All SAEs
- Health hazards that require measures
- Device deficiencies

The Sponsor-Investigator will evaluate SAEs with regard to causality and seriousness. Device deficiencies are assessed regarding their potential to lead to an SAE.

Pregnancies

Pregnancy is one of the exclusion criteria for the study. Participants that become pregnant during the study will need to report this to the study nurse/study physician. The participant will decide to complete the study or to proceed to early termination due to pregnancy.

Reporting to Authorities [ClinO Art. 42]:

In Category C studies it is the Investigator's responsibility to report serious adverse events in Switzerland, where it cannot be excluded that the events are attributable to the device under investigation, or to an intervention undertaken in the clinical trial to the Ethics Committee via BASEC within 7 days. The Sponsor-Investigator reports within the same timeline to Swissmedic (incl. events from abroad).

It is the Investigator's responsibility to report device deficiencies to the Ethics Committee via BASEC that could have led to serious adverse events if suitable action had not been taken, intervention had not been made, or circumstances had been less fortunate within 7 days. The Sponsor-Investigator reports within the same timeline to Swissmedic (incl. events from abroad) [ClinO Art. 42].

Health hazards that require measures are reported to Swissmedic and to the Ethics Committee via BASEC within 2 days [ClinO Art. 37].Periodic safety reporting:

In Category C studies a yearly safety update-report is submitted by the Investigator to the Ethics Committee and by the Sponsor-Investigator to Swissmedic.

10.1.4 Follow up of (Serious) Adverse Events

The follow-up will be defined by the PI at an individual study participant level.

11. STATISTICAL METHODS

11.1 Hypothesis

Null Hypothesis: It is not possible to identify reliable, well accepted biomarkers.

Alternative Hypothesis: It is possible to identify at least one reliable, well accepted biomarker for the following domains: a) vision b) cognition c) dexterity d) movement.

11.2 Determination of Sample Size

Due to the iterative study design, no formal sample size calculation was performed. The sample size of 30 PwMS and 30 HC was estimated based on published results of similar research in the field (Midaglia et al., J Med Internet Res. 2019).

11.3 Statistical criteria of termination of trial

There are no statistical discontinuation criteria for individual study participants.

11.4 Planned Analyses

11.4.1 Datasets to be analysed, analysis populations

All subjects who completed the study will be included in the primary analysis (per protocol analysis). An additional "intention-to-test" analysis comprising all eligible subjects will be performed as a sensitivity analysis.

11.4.2 Primary Analysis

The active tests will be selected using a stepwise selection strategy:

Step 1: Determination of reliability

For each active test of movement, vision and dexterity (e.g. "The Butler") the following analyses of reliability will be performed for each test feature (e.g. "normalized path length in anterior-posterior direction"):

a) Intra Class Correlation (ICC)

- Intra class correlation is frequently used as a measure for reliability.
- Measures the variance of test repetition between subjects in relation to the total variance across all repetitions and subjects.
- It is controversial, if guidelines for interpretation should be used at all, as ICC depends strongly on the analysed population (e.g. *Muller R, Buttner P. A critical discussion of intraclass correlation coefficients. Stat Med 1994 Dec 15-30;13(23-24):2465-76.)*
- Suggestion according to *Cicchetti V, Psychological Assessment,1994*
    - <0.4 - poor
    - 0.4-0.59 fair
    - 0.6-0.74 good
    - 0.75-1.0 excellent
- Based on these recommendations, **we will use an ICC ≥ 0.6 as indicator for "good" reliability.**
- A known issue with the ICC is that it tends to be low in very homogenous populations, even if the repeated measurements are stable.

b) The median coefficient of variation (CV)

- CV is calculated as the standard deviation of the data for each subject, divided by the subject's mean. Median CV will be used to summarise individual CVs.
- Focus on "within"-variance
- Not inferior in very homogenous populations
- Similar to the ICC, no general guidelines exist for interpretation of CV. Based on guidance in the field of bioanalytics, **we will use a median CV < 20% as a measure of sufficient reliability.** (see FDA-guidelines https://www.fda.gov/files/drugs/published/Bioanalytical-Method-Validation-Guidance-for-Industry.pdf
and EMA-guidelines https://www.ema.europa.eu/en/documents/scientific-guideline/guideline-bioanalytical-method-validation_en.pdf)

Step 2: Determination of user acceptance and adherence

a) Feedback on user acceptance will be captured with a feedback questionnaire and a semi-structured interview in week 6.

Every test and the overall impression of the App will be rated on a 5-step scale and comprise:

- Overall impression
- User friendliness
- Effort needed
- Meaningfulness for PwMS
- Willingness to perform the test in the future

A mean response of > 3 on a Likert Scale will be the minimal requirement for test selection.
b) Adherence will be calculated as percentage of completed repetitions divided by the total number of scheduled repetitions for each test. **The minimal adherence required for test selection will be 80%.**

Step 3: Test ranking and selection
For each test domain (vision, cognition, dexterity, movement) all tests that fulfill the minimal requirements for reliability in either ICC or CV, as well as user acceptance and adherence, will be ranked according to their performance in steps 1 and 2. Based on this ranking, tests for each domain will be selected to be further analysed in the validation study. If more than one test meets the minimal requirements, multiple tests per domain can be selected, if the different tests are of complementary clinical value.

11.4.3 Exploratory Analyses

The following exploratory analyses will be performed:
a) Potential differences between healthy controls and PwMS on a group level will be analysed with appropriate statistical tests, depending on data category and distribution (e.g. Wilcoxon Signed Rank Test). Although the study is not designed to detect statistically significant differences, results may be used to inform test selection for the validation study.

b) For each domain, correlations with established tests (e.g. "Catch-a-cloud" vs. Nine-Hole-Peg test) will be analysed in order to inform test selection for the validation study

11.4.4 Interim analyses
No interim analysis is planned due to the short study period (6 weeks).

11.4.5 Safety analysis

A safety analysis will be performed based on the reported AE and SAE after completion. This analysis will be performed by the CTU Basel in collaboration with the Principal Investigators.

11.4.6 Deviation(s) from the original statistical plan

Deviations from the original statistical plan will be described and justified in the final report.

11.5 Handling of missing data and drop-outs

Drop-outs will be replaced. Missing data will be handled by multiple imputation. A complete case analysis will be added as a sensitivity analysis.

12. QUALITY ASSURANCE AND CONTROL

Written SOPs and Working Instructions for all relevant study processes will be generated for all relevant study related processes and will be available on site. All study personnel will be trained before performing any activity related to study conduct. The respective documentation will be stored.

12.1 Data handling and record keeping / archiving

Trial Master File (TMF), Investigator Site File (ISF) and paper based participant related information will be archived in the University Hospital Basel.
All electronic records will be archived in a designated database at the University Hospital Basel.

12.1.1 Case Report Forms

Study data including demographic data will be obtained at V1,V2 and V3, coded and documented by CRF (paper version) and will thereafter be integrated in the eCRF provided by local CTU. Data obtained from paper based semi-structured interviews, will be entered by the study nurse.

12.1.2 Specification of source documents

Source data will be available at the study site and include all original documents relating to the study, as well as the medical treatment and medical history of the participants.
Source data are considered to consist of demographic data, visit dates, participation in the study and Informed Consent Forms, randomisation number, SAEs and AEs, test data, data from questionnaires and structured interviews, and the medical records.

12.1.3 Record keeping / archiving

All study data will be archived for a minimum of 10 years after study termination or premature termination of the clinical trial. Data will be stored at RC2NB (Spitalstrasse 2, 4056 Basel).

12.2 Data management

12.2.1 Data Management System

Study data including demographic data will be coded and documented by CRF (paper version) and will thereafter be integrated in the eCRF provided and stored by local CTU (secutrial). Data obtained from feedback questionnaires are integrated in the eCRF, data obtained from paper based semi-structured interviews will not be entered into eCRF and will be stored by the neuropsychologist of the team.
The DREAMS Application will collect sensor data from participants' smartphones while they are performing the activities. When a study subject completes a task on his or her

smartphone, the DREAMS App sends the compressed and encrypted raw data to the DREAMS cloud for validation and processing. Data from the participants' wearable (FitBit) is collected via that wearable via an API (interface) of the manufacturer (FitBit). Healios and FitBit have established an interface through which the data is transferred securely from FitBit to the central database. The data captured via the App is directly stored in a table structure that reflects the structure of the App and the activities. Each of the datasets is linked to the user via a coded patient identifier, along with time and date stamps.
The central database (DREAMS cloud) is a MySQL database management solution that is hosted in an Amazon WebServices (AWS) environment. During the study, the data is managed by Healios database administration. The DREAMS cloud is internally organized by a multitude of microservices in a "virtual private cloud" (VPC) hosted on a server farm by Amazon Web Services (AWS) in Frankfurt, Germany as primary zone. Dublin, Ireland is set-up as secondary zone with the objective of achieving high availability. The system architecture is divided into separate, independent microservices, to increase flexibility, scalability and reusability of codes. The DREAMS cloud includes an administrator service as a gateway, a queue service that controls asynchronous jobs in order to reduce the time of response of the system, a user data service to manage the user data, a manager service to manage transactional information related to the tests ("challenges"), e.g. schedule, completion, reporting, and a validator service that validates and parses the raw data and generates a new dataset, called "pre-processed" data. Finally, a processor service runs the different algorithms over the pre-processed data and will generate the processed data ("results", e.g. features/metrics). Each stage of the data (raw, pre-processed and processed/results) will be stored in a database called "Precard DB".
The DREAMS platform will send each dataset (raw, pre-processed and processed data) daily to a data warehouse hosted on local premises by University Hospital Basel IT department, where the data is mirrored.

12.2.2 Data security, access and back-up

The DREAMS Application is downloaded on the personal smartphone of study participants by a trained study nurse or physician who also assigns each participant a unique study code. Neither the DREAMS App nor the RC2NB main partner Healios GmbH have access to any personal (health) information of study participants. The lookup-table of study codes and patient IDs remains with RC2NB and is not accessible to other parties. The solution is hosted on a fully secured and encrypted virtual private network (VPN) Amazon WebServices (AWS) environment. To ensure high security standards, AWS functionalities are used, such as logically isolated virtual private networks, database encryption, user and authentication management, activity logging, DDoS protection as well as daily-encrypted backups. Data transmission between the App cloud and University Hospital IT and is encrypted via HTTPS with AWS Certificate Manager.
Access to data is limited to the designated data manager at Healios GmbH, and authorized study personnel who require the data to fulfil their duties within the scope of the research project
The servers are located in Frankfurt, Germany (primary) and Dublin, Ireland (secondary). All the databases have an integrated back-up system for regular backups. In addition, the DREAMS platform will send daily mirrors to a data warehouse hosted on local premises by University Hospital Basel IT department.

12.2.3 Analysis and archiving

Data will be extracted from the data warehouse upon study completion and analysed by designated data analysts/biostatisticians at RC2NB and CTU Basel. Researchers from University of Basel (sciCORE) and 3rd party collaborators can also receive copies of pseudonymized data sets as foreseen in respective data sharing agreements in accordance with applicable data privacy legislation. Study data will be stored in a data warehouse hosted by University Hospital Basel IT department. All study data will be archived for a minimum of 10 years after study termination or premature termination of the clinical trial.

12.2.4 Electronic and central data validation

Procedures are in place to monitor that sensor data are captured and stored accurately and completely during the study and data management procedures are in place. Validation and processing include outlier detection, imputation of missing values, noise reduction and data analysis to extract features/metrics. The solution has completeness and validation checks to support quality of the manually entered data, e.g. when completing validated scales.

12.3 Monitoring

Monitoring visits by the CTU Basel will be performed prior to study start and once after study completion, see separate monitoring plan by CTU.
Source data will be accessible to monitors and questions will be answered during monitoring.

12.4 Audits and Inspections

External Auditing is not planned for this study. However, if an external audit/inspection should be requested by a regulatory body study, documentation and source data will be made available to auditors/inspectors.

12.5 Confidentiality, Data Protection

Protocol, full dataset, statistical code and algorithms will be accessible by the Principal Investigators, the statistical as well as the data management teams during and after the study.

Direct access to source documents will be permitted for purposes of monitoring, audits and inspections.

12.6 Storage of biological material and related health data

No biological material is stored for this study.

13. PUBLICATION AND DISSEMINATION POLICY

A scientific publication will be prepared by the PI's of this study and is planned for 2021. The protection of trade secrets regarding the Medical Device will be respected.

14. FUNDING AND SUPPORT

This project is funded by Innosuisse no. 33535.1 IP-ICT.

14.1 Funding

This project is funded by Innosuisse no. 33535.1 IP-ICT.

14.2 Other Support

There is no additional support.

15. INSURANCE

Insurance will be provided by the Sponsor. A copy of the certificate is filed in the investigator site file and the trial master file.

16. REFERENCES

1. Declaration of Helsinki, Version October 2013, (http://www.wma.net/en/30publications/10policies/b3/index.html)
2. International Conference on Harmonization (ICH, 1996) E6 Guideline for Good Clinical Practice. (http://www.ich.org/fileadmin/Public_Web_Site/ICH_Products/Guidelines/Efficacy/E6/E6_R2__Step_4.pdf)
3. International Conference on Harmonization (ICH, 1997) E8 Guideline: General Considerations for Clinical Trials http://www.ich.org/fileadmin/Public_Web_Site/ICH_Products/Guidelines/Efficacy/E8/Step4/E8_Guideline.pdf)
4. Humanforschungsgesetz, HFG Bundesgesetz über die Forschung am Menschen (Bundesgesetz über die Forschung am Menschen, HFG) vom 30. September 2011/ Loi fédérale relative à la recherche sur l'être humain (loi relative à la recherche sur l'être humain, LRH) du 30 septembre 2011 / Legge federale concernente la ricerca sull'essere umano (Legge sulla ricerca umana, LRUm) del 30 settembre 2011
5. Verordnung über klinische Versuche in der Humanforschung (Verordnung über klinische Versuche, KlinV) vom 20. September 2013 / Ordonnance sur les essais cliniques dans le cadre de la recherche sur l'être humain (Ordonnance sur les essais cliniques, OClin) du 20 septembre 2013. Ordinanza sulle sperimentazioni cliniche nella ricerca umana (Ordinanza sulle sperimentazioni cliniche, OSRUm) del 20 settembre 2013
6. Heilmittelgesetz, HMG Bundesgesetz über Arzneimittel und Medizinprodukte (Heilmittelgesetz, HMG) vom 15. Dezember 2000 / Loi fédérale sur les médicaments et les dispositifs médicaux (Loi sur les produits thérapeutiques, LPT) du 15 décembre 2000 / Legge federale sui medicamenti e i dispositivi medici (Legge sugli agenti terapeutici, LATer)
7. ISO 14155:2011 Clinical investigation of medical devices for human subjects -- Good clinical practice (www.iso.org)
8. ISO 10993 Biological evaluation of medical devices (www.iso.org)
9. MEDDEV 2.7/3 revision 3, May 2015
10. Medizinprodukteverordnung (MepV) vom 17. Oktober 2001 / Ordonnance sur les dispositifs médicaux (ODim) du 17 octobre 2001 / Ordinanza relativa ai dispositivi medici (ODmed) del 17 ottobre 2001
11. WHO, International Clinical Trials Registry Platform (ICTRP) (http://www.who.int/ictrp/en/)
12. European regulation on medical devices 2017/745.
13. Strahlenschutzverordnung (StSV) vom 26. April 2017 / Ordonnance sur la radioprotection (ORaP) du 26 avril 2017 / Ordinanza sulla radioprotezione (ORaP) del 26 aprile 2017.
14. Lassmann H, Bruck W, Luccinetti CF. The immunopathology of multiple sclerosis: an overview. *Brain pathology*. 2007; 17(2): 210-218.
15. Confavreux C, Vukusic S, Moreau T, Adeleine P. Relapses and progression of disability in multiple sclerosis. *The New England journal of medicine*. 2000; 343(29): 1430-1438.
16. Thompson AJ, Banwell BL, Barkhof F, et al. Diagnosis of multiple sclerosis: 2017 revisions of the McDonald criteria. *The Lancet. Neurology*. 2018;17(2):162-173.

17. Perry B, Herrington W, Goldsack JC, et al. Use of Mobile Devices to Measure Outcomes in Clinical Research, 2010-2016: A Systematic Literature Review. *Digit Biomark.* 2018; 2(1): 11-30.
18. Maillart E, Labauge P, Cohen M, et al. MSCopilot, a new multiple sclerosis self-assessment digital solution: results of a comparative study versus standard tests. *European journal of neurology: the official journal of the European Federation of Neurological Societies.* 2019.

17. APPENDICES

17.1 IMP: IB or SPC

Not applicable

17.2 Medical Devices: IB (according to ISO 14155)

See separate document: DREAMS R1.4 Investigators Brochure v1.0_signed

17.3 Medical Devices: Assurance of producer

See separate document: Manufacturer Assurance Feasiblity Study – v.1.0_signed

17.4 Medical Devices: List of norms (vollständig eingehaltene, teilweise eingehaltene)

Applicable Standards:

- Clinical management: ISO 14155
- Risk management: ISO 14971
    - Software related: cybersecurity, deployment, data quality, Software Of Unknown Provenance (SOUP)
- SW: IEC 62304 & 82304
- Usability: IEC 62366
- Quality management: ISO 13485

Table 12

	Wearable	Smartphone	DREAMS App	Central dBM	Algorithms	Dashboard
MD	No	No	Yes, SaMD	No	Yes, SaMD	Yes, SaMD
Risks	Deployment<br>Cybersecurity<br>Data quality	Deployment<br>Cybersecurity<br>Data quality	Deployment<br>Cybersecurity	Deployment<br>Cybersecurity<br>SOUP	Deployment<br>Cybersecurity<br>SOUP	Deployment<br>Cybersecurity
V&V	Included in RM<br>Config. Mmt.	Included in RM<br>Config. Mmt.	RM ISO 14971<br>Usability IEC 62366<br>SW IEC 62304<br>SW IEC 82304	SW IEC 62304	RM ISO 14971<br>SW IEC 62304<br>**Clinical ISO 14155**	SW 62304<br>Usability IEC 62366<br>**Clinical ISO 14155**

Table 12: List of norms for Medical Devices

17.5 APP contents

17.5.1. Questionnaires (in german)

17.5.1.1. MSIS-29

	1 Gar nicht	2 Ein bisschen	3 Mässig	4 Ziemlich	5 Sehr
Wie schwer fiel es Ihnen in den letzten zwei Wochen …					
1. körperlich anstrengende Dinge zu tun?	☐	☐	☐	☐	☐
2. Dinge fest anzufassen (z.B. Hahn aufdrehen)?	☐	☐	☐	☐	☐
3. Dinge zu tragen?	☐	☐	☐	☐	☐
Hatten Sie in den letzten zwei Wochen …					
4. Probleme mit dem Gleichgewicht?	☐	☐	☐	☐	☐
5. Schwierigkeiten, sich in der Wohnung zu bewegen?	☐	☐	☐	☐	☐
6. das Gefühl ungeschickt zu sein?	☐	☐	☐	☐	☐
7. ein Steifigkeitsgefühl?	☐	☐	☐	☐	☐
8. schwere Arme und/oder Beine?	☐	☐	☐	☐	☐
9. Zittern der Arme oder Beine?	☐	☐	☐	☐	☐
10. Krämpfe der Extremitäten?	☐	☐	☐	☐	☐
11. das Gefühl, dass ihr Körper nicht tat, was sie wollten?	☐	☐	☐	☐	☐
12. Beeinträchtigung im sozialen und Freizeitleben zu Hause?	☐	☐	☐	☐	☐
13. Probleme mit den Händen bei Alltagstätigkeiten?	☐	☐	☐	☐	☐
14. Probleme sich fortzubewegen (Auto, Bus, Taxis, Zug)?	☐	☐	☐	☐	☐
15. länger gebraucht, Dinge zu tun?	☐	☐	☐	☐	☐
16. Schwierigkeiten, Dinge spontan zu machen?	☐	☐	☐	☐	☐
17. das Gefühl, ganz schnell zur Toilette zu müssen?	☐	☐	☐	☐	☐
18. sich allgemein unwohl gefühlt?	☐	☐	☐	☐	☐
19. Schlafprobleme?	☐	☐	☐	☐	☐
20. sich geistig/mental müde gefühlt?	☐	☐	☐	☐	☐
21. Sorgen bezogen auf ihre MS?	☐	☐	☐	☐	☐
22. sich angespannt und ängstlich gefühlt?	☐	☐	☐	☐	☐
23. sich ungeduldig und aufbrausend gefühlt?	☐	☐	☐	☐	☐

24. Konzentrationsprobleme?	☐	☐	☐	☐	☐
25. keine Zuversicht?	☐	☐	☐	☐	☐
26. sich traurig/depressiv gefühlt?	☐	☐	☐	☐	☐
Waren Sie in den letzten zwei Wochen ...					
27. davon abhängig, dass andere Dinge für Sie erledigten?	☐	☐	☐	☐	☐
28. gezwungen, zu Hause zu bleiben?	☐	☐	☐	☐	☐
29. gezwungen die Zeit für Arbeit oder Alltagsaktivitäten einzuschränken?	☐	☐	☐	☐	☐

17.5.1.2. MSWS-12

	1 Ueber-haupt nicht	2 Ein wenig	3 Mässig	4 Ziemlich stark	5 Extrem stark
Wie stark hat Ihre MS in den letzten 14 Tagen:					
1. Ihre Gehfähigkeit eingeschränkt?	☐	☐	☐	☐	☐
2. Ihre Lauffähigkeit eingeschränkt?	☐	☐	☐	☐	☐
3. Ihre Fähigkeit, Treppen hinauf oder herunter zu gehen, eingeschränkt?	☐	☐	☐	☐	☐
4. es Ihnen erschwert, Dinge im Stehen zu tun?	☐	☐	☐	☐	☐
5. Ihr Gleichgewicht beim Stehen oder Gehen eingeschränkt?	☐	☐	☐	☐	☐
6. eingeschränkt, wie weit Sie gehen konnten?	☐	☐	☐	☐	☐
7. die von Ihnen zum Gehen benötigte Anstrengung erhöht?	☐	☐	☐	☐	☐
8. es für Sie erforderlich gemacht, sich abzustützen, wenn Sie drinnen gehen, z.B. sich an Möbeln festzuhalten, einen Stock zu verwenden etc.?	☐	☐	☐	☐	☐
9. es für Sie erforderlich gemacht, sich abzustützen, wenn Sie draussen gehen, z.B. einen Stock oder einen Gehrahmen zu verwenden?	☐	☐	☐	☐	☐
10. Ihr Gehen verlangsamt?	☐	☐	☐	☐	☐

11. sich darauf ausgewirkt, wie problemlos und gleichmässig Sie gehen?	☐	☐	☐	☐	☐
12. dazu geführt, dass Sie sich auf Ihr Gehen konzentrieren?	☐	☐	☐	☐	☐

17.5.1.3. Fatigue-Skala

	1 Ich stimme gar nicht zu	2	3	4	5	6	7 Ich stimme vollkommen zu
Ich finde, dass in den letzten 14 Tagen folgendes zutraf:							
1. Ich bin weniger motiviert, wenn ich müde bin.	☐	☐	☐	☐	☐	☐	☐
2. Körperliche Bewegung macht mich müde.	☐	☐	☐	☐	☐	☐	☐
3. Ich ermüde rasch.	☐	☐	☐	☐	☐	☐	☐
4. Meine Müdigkeit beeinträchtigt meine körperliche Leistungsfähigkeit	☐	☐	☐	☐	☐	☐	☐
5. Meine Müdigkeit bereitet mir oft Probleme.	☐	☐	☐	☐	☐	☐	☐
6. Meine Müdigkeit verhindert längerdauernde körperliche Tätigkeiten	☐	☐	☐	☐	☐	☐	☐
7. Meine Müdigkeit beeinträchtigt mich, gewisse Pflichten und Verantwortungen zu erfüllen.	☐	☐	☐	☐	☐	☐	☐
8. Meine Müdigkeit gehört zu den Beschwerden, die mich am meisten behindern.	☐	☐	☐	☐	☐	☐	☐
9. Meine Müdigkeit beeinträchtigt meine Arbeit, meine Familie	☐	☐	☐	☐	☐	☐	☐

oder mein soziales Leben.				

17.5.1.4. MS Symptom Tracker

	1 Keine	2 Leicht	3 Mittel	4 Schwer
Haben Sie in den letzten zwei Wochen eines der folgenden Symptome erlebt? Wenn ja, wie schwerwiegend waren diese Symptome?				
1. Müdigkeit	☐	☐	☐	☐
2. Schmerzen	☐	☐	☐	☐
3. Blasenprobleme	☐	☐	☐	☐
4. Darmprobleme	☐	☐	☐	☐
5. Taubheitsgefühl	☐	☐	☐	☐
6. Kognitive Beeinträchtigung	☐	☐	☐	☐
7. Schwindel	☐	☐	☐	☐
8. Sexualfunktionsstörung	☐	☐	☐	☐
9. Probleme beim Gehen	☐	☐	☐	☐
10. Gleichgewichtsprobleme	☐	☐	☐	☐
11. Koordinationsstörung	☐	☐	☐	☐
12. Gedächtnisprobleme	☐	☐	☐	☐
13. Sehstörung	☐	☐	☐	☐
14. Muskelkrämpfe	☐	☐	☐	☐
15. Steifheit der Muskeln	☐	☐	☐	☐
16. Schwäche	☐	☐	☐	☐

17.5.1.5. Relapse Protocol

The following questions will be asked, if participant is reporting a relapse:

	Ja			Nein
1. Haben Sie eine Schwäche oder ein Taubheitsgefühl bemerkt?	leicht	mittel	schwer	
Linker Arm/Hand	☐	☐	☐	☐
Rechter Arm/Hand	☐	☐	☐	☐
Linkes Bein/Fuss	☐	☐	☐	☐
Rechtes Bein/Fuss	☐	☐	☐	☐
2. Haben Sie Probleme mit dem Gleichgewicht oder der Koordination bemerkt?				
	☐			☐
3. Haben Sie eine Sehstörung oder Missempfindung in den Augen bemerkt?		Linkes Auge		
	☐	☐	☐	☐
		Rechtes Auge		
	☐	☐	☐	☐
4. Haben Sie Doppelbilder bemerkt?				
	☐			☐
5. Blasen- oder Darmprobleme				
a. Harndrang (Das Gefühl, die Blase sofort entleeren zu müssen)	☐			☐
b. Häufigkeit (vermehrte Häufigkeit des Wasserlassens)	☐			☐
c. Nächtliches Wasserlassen (Häufiges Wasserlassen in der Nacht)	☐			☐
d. Inkontinenz (unwillkürlicher Harnverlust)	☐			☐
e. Schwierigkeiten das Wasserlassen zu beginnen)	☐			☐
f. Verstopfung (Harte Stuhlgänge)	☐			☐
g. Durchfall (täglich dünner, flüssiger oder wässriger Stuhlgang)	☐			☐
6. Haben Sie Probleme mit der Kognition bemerkt?				
a. Probleme mit dem Gedächtnis	☐			☐
b. Probleme mit der Konzen-	☐			☐

tration	

The following information will be displayed: «Ihre Schubinformation wurde erfolgreich gespeichert. Wir werden den Verlauf in 7 und 14 Tagen noch einmal erfragen».

7 Days after the patient reported a relapse, they will be asked how they are doing:

	Ja			Nein
7. Haben Sie sich erholt?	Ja, vollständig	Ja, teilweise		Nein
	☐	☐		☐
8. Wie lange hat die Erholung gedauert?	1-3 Tage	3-7 Tage	Ich habe mich noch nicht ganz erholt	
	☐	☐	☐	
9. Haben Sie Kortikosteroide («Kortison») erhalten?	Ja			Nein
	☐			☐
10. Bemerkten Sie Beeinträchtigungen bezüglich (alle zutreffenden auswählen)	Ja			Nein
a. Gehen	☐			☐
b. Sprechen	☐			☐
c. Tremor	☐			☐
d. Fatigue (Erschöpfung)	☐			☐
e. Keine	☐			☐

14 Days after the patient reported a relapse, they will be asked how they are doing:

	Ja			Nein
11. Haben Sie sich erholt?	Ja, vollständig	Ja, teilweise		Nein
	☐	☐		☐
12. Wie lange hat die Erholung gedauert?	1-3 Tage	3-7 Tage	7-10 Tage	10-14 Tage

	☐	☐	☐	☐
13. Haben Sie Kortikosteroide («Kortison») erhalten?	Ja			Nein
	☐			☐
14. Bemerkten Sie Beeinträchtigungen bezüglich (alle zutreffenden auswählen)	Ja			Nein
a. Gehen	☐			☐
b. Sprechen	☐			☐
c. Tremor	☐			☐
d. Fatigue (Erschöpfung)	☐			☐
e. Keine	☐			☐

17.6 Risk Management

The Risk Score is the product of probability and impact of a specified risk and the definitions used are the following:

Table 13:

Probability	Score	Definition
Remote	1	Probability of less than 10%
Unlikely	2	Probability between 10% and 35%
Possible	3	Probability between 36% to 64%
Likely	4	Probability 65% to 90%
Certain	5	Probability above 90%
**Impact**	**Score**	**Definition**
Insignificant	1	Easily handled within the normal course of operations with no additional costs
Minor	2	Some disruption within the normal functions. Manageable risk with minimum estimated cost
Moderate	3	Immediate time/resource reallocation will be necessary with a moderate estimated cost
Major	4	Operations are severely disrupted and significant risk of failure to part of the business is possible
Critical	5	Significant going concerns exists with the business and the risk is classified as critical

Table 13: Defining Probability and Impact of the risk score

The Risk Score in numbers and defining risk definitions (low, low medium, medium, medium high and high) see graph 4.

Graph 4

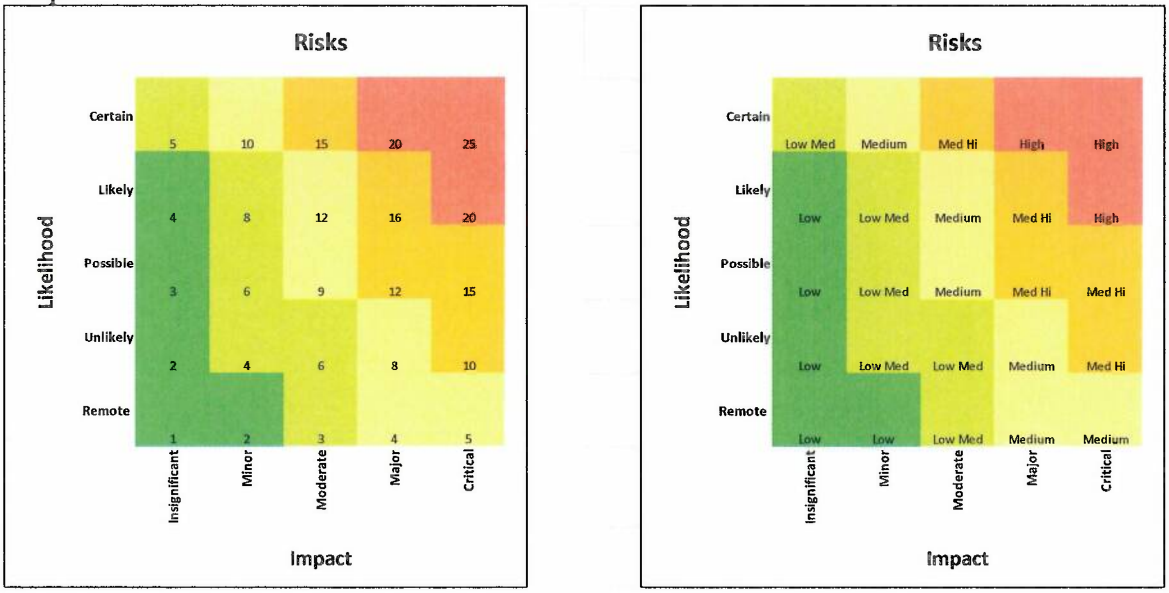

Graph 4: Risk scores and risk definitions

Table 14

Risk Number	Risk	Before Measure					After Defined Measures			
		Probability	Impact	Risk Score	Risk	Measure	Probability	Impact	Risk Score	Risk
1	Strategy Healios (Main partner)	unlikely	critical	10	Medium High	The company has committed their strategy to digital biomarkers and this project. Agreement between cooperation partners with smart deliverables. Be proactive with stakeholders about on-going strategic challenges	unlikely	minor	4	Low Med
2	Financial Plan Healios	possible	moderate	9	Medium	Growing company with investors for a couple of years. Additional funding through RC2NB	remote	moderate	3	Low Med
3	Competitive Landscape	certain	moderate	15	Medium High	Assure excellent quality and adhere to time schedule of study in order to present best solution at time of launch	remote	critical	5	Medium
4	Knowledge Transfer Healios/USB	possible	moderate	9	Medium	Weekly team meetings with the core development team. Manpower has already being adapted on both sides	remote	moderate	3	Low Med
5	Technology Aspects	likely	moderate	12	Medium	DREAMS relies on the quality of the data collected and therefore may be used with a vast array of consumer devices. The company will ensure that the important information will be sensor based and not device based. Also the company can adapt the	remote	minor	2	Low

						app during the study to optimize data capture quality. Healios adaptability will enable address technological changes				
6	Change in laws/regulatory aspects	unlikely	moderate	6	Low Med	Regular exchange with local CTU and specialised consultancies to ensure legal team remains on top of any change in privacy law.	remote	moderate	3	Low Med
7	Approval by regulatory authorities (refusal)	unlikely	critical	10	Medium High	Highly experienced team at RC2NB ensures quality of documents. The study protocol and all ICF's will be reviewed by the local Clinical Trial Unit to ensure and double check the quality and completeness of the documentation	remote	critical	5	Medium
8	Approval by regulatory authorities (delay)	likely	moderate	12	Medium	Highly experienced team at RC2NB ensures quality of documents. The study protocol and all ICF's will be reviewed by the local Clinical Trial Unit to ensure and double check the quality and completeness of the documentation	possible	moderate	9	Medium
9	Data Analysis	possible	critical	15	Medium High	Cooperation with potential partners for complex data analysis was discussed. We established a cooperation with sciCORE (https://scicore.unibas.ch) and have contact to other interested partners for additional analysis. We	unlikely	major	8	Medium

						made sure to have the required expertise in Basel.				
10	Strategy Plan Neurology Dep. USB	possible	major	12	Medium High	The establishment of RC2NB (CEO: Prof. Ludwig Kappos) guarantees the team working on the project independently and over a period for at least 6 years. Discussions with the potential future head of Department of Neurology (retirement of Prof. Kappos planned for October 2020) were performed to make sure, that the cooperation with the Department of Neurology remains close.	unlikely	major	8	Medium
11	Study Design	possible	major	12	Medium High	Highly experienced team at RC2NB under the lead of Prof. Ludwig Kappos ensures the quality of the study protocol. Advice of CTU has been sought. The study protocol was already presented to and discussed with the clinical expert of Tüv Süd. Results from the Feasibility study will be used to adapt the design of Validation study 1 accordingly.	unlikely	major	8	Medium
12	Recruitment	possible	critical	9	Medium High	Existing cohort of patients with MS motivated to take part in clinical studies. Controlling of recruitment performance will be performed and the strategy of recruitment will be	unlikely	moderate	6	Low Med

						adapted accordingly (extension to other cohort centres if needed). The study has been presented at patient events and patients already interested to participate to the study have been listed.				
13	Reliable Biomarkers	possible	critical	15	Medium High	A careful pre-selection of biomarkers that seem to be feasible and measurable was designed by the team. Error analysis and technical trouble shooting is performed continuously. Any effort is made to produce a set of reproducible biomarkers at the end of the feasibility phase.	unlikely	critical	10	Medium High
14	Resources and Staffing RC2NB/ Healios	possible	moderate	9	Medium	Manpower has already been adapted for both teams. Will be further adapted along the needs.	unlikely	moderate	6	Low Med
15	Statistical Analysis Plan	possible	major	12	Medium High	Professional input of local Clinical Trial Unit with years of experience in conduct of clinical trials as well as input from two independent statisticians has been sought.	unlikely	major	8	Medium
16	Patent Application	possible	major	12	Medium High	Is followed continuously by main partner. Specific manpower is dedicated to this topic already.	unlikely	major	8	Medium
17	Failure to meet endpoints	possible	major	12	Medium High	Advanced study design that allows for a flexible set of various single biomarkers to be analysed.	unlikely	major	8	Medium

						Cluster analysis of biomarkers to determine various subgroups. Use of endpoints reflecting different aspects of disease progression (clinical, imaging, body fluid biomarkers) ranging from very sensitive to change to very robust. Patterns of biomarkers will be analysed, allowing to pursue combinations of the ones with best performance to either discriminate between patient subgroups and correlate with established markers of disease progression. These patterns may change according to the different outcomes. Pattern of biomarkers identified that allows for clustering of patient groups and correlates with established markers of disease progression.				
**Total Risk Score**				**191**					**104**	

Table 14: Risk Management overview - identified risks and the impact of defined measures
