# Supplementary material for: Assessment of cognitive performance in multiple sclerosis using smartphone-based training games: a feasibility study
Source: J Neurol. 2023 Mar 23;270(7):3451–63. doi: 10.1007/s00415-023-11671-9 (PMC10267276; doi:10.1007/s00415-023-11671-9)
Supplement: Supplementary file 5 — Supplementary file5 (PDF 101 KB) [file 415_2023_11671_MOESM5_ESM.pdf]

Supplementary Material Table S1:

Description of cognitive games and measures used for statistical analyses

Cognitive Game	Game description	Measures
Word Hunt	A grid of letters is shown. The subject must search for words, which are hidden vertically, horizontally, and diagonally in the grid.	Completion time in seconds
Spin Cycle	While a rotating circle of geometrical shapes is shown, the subject must memorize their shape, color, size, direction, and rotation speed. Thereafter another rotating circle is presented, and the subject is asked if one of the properties has changed.	Difficulty-level reached (Range: 0-6)
Zap Gap	Multiple differently colored lines are shown. Colored spheres “drop down” the lines. If the color of the sphere and line don’t match, the user must tap on it at the right moment.	Difficulty-level reached (Range: 0-11)
Face Switch	Multiple images with a man and a woman are shown and questions about details of the persons (e.g., does the woman wear glasses? Does the man smile?) must be answered as quickly as possible.	Number of successful rounds
Rush Back	Different shapes are successively shown, and the subject must continuously decide whether the currently shown one matches the previous one.	Number of successful rounds
Baggage Claim	The user must memorize names of cities which then have to be selected after an interference task (e.g. sorting names alphabetically), from a given list.	Difficulty-level reached (Range: 0-14)
Perilous Path	A grid with red “bombs” must be memorized in order to draw a path between 2 points without touching the bombs after the whole grid has been hidden.	Difficulty-level reached (Range: 0-16)
Puzzle Blox	Subjects must reconstruct a presented geometrical figure, by eliminating parts of a larger figure.	Difficulty-level reached (Range: 0-8)
Must Sort	The subject must sort cards (left or right), according to their symbol/color as fast as possible.	Number of successful rounds
Low Pop	The user must tap items in ascending order as fast as possible.	Difficulty-level reached
