# Supplementary material for: Assessment of cognitive performance in multiple sclerosis using smartphone-based training games: a feasibility study
Source: J Neurol. 2023 Mar 23;270(7):3451–63. doi: 10.1007/s00415-023-11671-9 (PMC10267276; doi:10.1007/s00415-023-11671-9)
Supplement: Supplementary file 6 — Supplementary file6 (PDF 118 KB) [file 415_2023_11671_MOESM6_ESM.pdf]

Supplementary Material Table S2:

Mean acceptance ratings on Likert scale (1-5) by group and topic (HC vs pwMS)

Game	Overall impression Mean (SD)			Difficulty Mean (SD)			Future Use Mean (SD)		
	**HC (n=29)**	**pwMS (n=30)**	**Rank biserial correlation**	**HC (n=29)**	**pwMS (n=30)**	**Rank biserial correlation**	**HC (n=29)**	**pwMS (n=30)**	**Rank biserial correlation**
Word Hunt	4.84 (±0.45)	4.97 (±0.18)	$\|r_{rb}\|$= -.180	4.39 (±1.15)	4.77 (±0.43)	$\|r_{rb}\|$= -.122	4.71 (±0.82)	4.84 (±0.82)	$\|r_{rb}\|$= -.014
Spin Cycle	4.29 (±0.90)	4.00 (±1.06)	$\|r_{rb}\|$= .139	4.16 (±0.93)	4.23 (±0.80)	$\|r_{rb}\|$= -.016	4.13 (±1.09)	4.16 (±1.09)	$\|r_{rb}\|$= -.051
Zap Gap	4.03 (±1.14)	4.32 (±1.01)	$\|r_{rb}\|$= -.138	4.06 (±1.06)	4.19 (±0.95)	$\|r_{rb}\|$= -.045	4.00 (±1.06)	4.32 (±1.06)	$\|r_{rb}\|$= -.161
Face Switch	4.68 (±0.60)	4.68 (±0.75)	$\|r_{rb}\|$= -.059	4.35 (±1.08)	4.45 (±0.89)	$\|r_{rb}\|$= -.002	4.50 (±1.01)	4.52 (±1.01)	$\|r_{rb}\|$= .022
Rush Back	4.74 (±0.51)	4.58 (±0.76)	$\|r_{rb}\|$= .088	4.23 (±1.20)	4.42 (±0.81)	$\|r_{rb}\|$= -.019	4.48 (±1.06)	4.35 (±1.06)	$\|r_{rb}\|$= .102
Baggage Claim	4.84 (±0.45)	4.58 (±0.92)	$\|r_{rb}\|$= .140	4.32 (±1.01)	4.65 (±0.80)	$\|r_{rb}\|$= -.177	4.61 (±0.76)	4.58 (±0.76)	$\|r_{rb}\|$= 025
Perilous Path	4.74 (±0.77)	4.94 (±0.25)	$\|r_{rb}\|$= -.156	4.45 (±0.93)	4.67 (±0.55)	$\|r_{rb}\|$= -.087	4.68 (±0.91)	4.77 (±0.91)	$\|r_{rb}\|$= .063
Puzzle Blox	4.58 (±0.96)	4.71 (±0.59)	$\|r_{rb}\|$= -.014	4.42 (±1.03)	4.55 (±0.62)	$\|r_{rb}\|$= .019	4.55 (±0.89)	4.61 (±0.89)	$\|r_{rb}\|$= .038
Must Sort	4.68 (±0.60)	4.74 (±0.68)	$\|r_{rb}\|$= -.108	4.26 (±1.12)	4.39 (±1.09)	$\|r_{rb}\|$= -.070	4.42 (±1.12)	4.52 (±1.12)	$\|r_{rb}\|$= .034
Low Pop	4.77 (±0.56)	4.90 (±0.30)	$\|r_{rb}\|$= -.105	4.48 (±1.00)	4.65 (±0.71)	$\|r_{rb}\|$= -.053	4.71 (±0.78)	4.58 (±0.78)	$\|r_{rb}\|$= .178
